# Overexpression of a Novel Arabidopsis Gene *SUPA* Leads to Various Morphological and Abiotic Stress Tolerance Alternations in Arabidopsis and Poplar

**DOI:** 10.3389/fpls.2020.560985

**Published:** 2020-11-12

**Authors:** Changyang Cai, Wenjia Wang, Shanwen Ye, Zhiliang Zhang, Wensha Ding, Mengqi Xiang, Chu Wu, Qiang Zhu

**Affiliations:** Basic Forestry and Proteomics Center (BFPC), Fujian Provincial Key Laboratory of Haixia Applied Plant Systems Biology, Haixia Institute of Science and Technology, College of Forestry, Fujian Agriculture and Forestry University, Fujian, China

**Keywords:** abiotic stress, peroxisome, ROS, poplar, Arabidopsis, morphological alternations

## Abstract

With the development of sequencing technology, the availability of genome data is rapidly increasing, while functional annotation of genes largely lags behind. In Arabidopsis, the functions of nearly half of the proteins are unknown and this remains one of the main challenges in current biological research. In an attempt to identify novel and rapid abiotic stress responsive genes, a number of salt-up (*SUP*) regulated genes were isolated by analyzing the public transcriptomic data, and one of them, *SUPA*, was characterized in this study. The expression of *SUPA* transcripts was rapidly up-regulated by various abiotic stress factors (<15 min), and SUPA protein is mainly localized in the peroxisome. Overexpression of *SUPA* in Arabidopsis leads to the elevated accumulation of reactive oxygen species (ROS), strong morphological changes and alternations in abiotic stress tolerance. The transcriptome analysis showed changes in expression of genes involved in stress response and plant development. Interestingly, ectopic overexpression of *SUPA* in poplar leads to a dwarf phenotype with severely curved leaves and changes in the plant tolerance of abiotic stresses. Our study reinforces the potential roles of *SUPA* in normal plant growth and the abiotic stress response.

## Introduction

As a sessile organism, plants have developed complicated survival mechanisms at physiological, molecular and cellular levels to adapt to the harmful conditions such as drought, cold and salinity. Upon stress, plants rapidly respond within several minutes which provokes global changes in many metabolic molecular and biochemical pathways, such as ROS, calcium waves, electric signals and hydraulic waves (Kollist et al., 2018). Through large transcriptome analyses, it was revealed that in general two groups of genes respond to abiotic stress stimuli, namely regulatory proteins and functional proteins (Shinozaki et al., 2003; Shinozaki and Yamaguchi-Shinozaki, 2006). Regulatory proteins such as various transcription factors, protein kinases, protein phosphatases or proteinases were normally activated rapidly (Lee and Luan, 2012; Khan et al., 2016; Zhu, 2016). Several transcription factor families were involved in the rapid response of plants to different stresses, such as *ethylene- responsive element binding factor/APETALA2* (*ERF/AP2*), *WRKY*, heat shock factors (*HSFs*), and *MYB* (Zhu, 2016). *DREB* (dehydration responsive element binding) family genes which belong to the *AP2/ERF* family and govern the expression of many stress-inducible target genes via a specific *cis*-acting element, the dehydration responsive element/C-repeat (DRE/CRT; A/GCCGAC), are well characterized players in plant responses to salt, drought or freezing conditions (Chew and Halliday, 2011). Upon stress, *CBF1/DREB1B* and *CBF3/DREB1A* transcripts start to accumulate 15 min after cold treatment and rapidly increase over the next 90 min (Gilmour et al., 1998; Novillo et al., 2004). *WRKY25* and *WRKY33* are responsive to salt treatment, and were considered as positive regulators in response to salt stress (Jiang and Deyholos, 2009). These genes normally act as the master regulators that positively or negatively regulate the expression of a wide range of genes involved in the pathways for plant adaption and survival, and the overexpression of the rapid response factors in plants that significantly change plant tolerance to salt (Jiang and Deyholos, 2009; Lata and Prasad, 2011).

The Reactive Oxygen Species (ROS) accumulation was always enhanced when plants suffer environmental constraints (Hossain et al., 2015; Del Rio and Lopez-Huertas, 2016). ROS which is mainly produced in chloroplasts, mitochondria, and peroxisomes is associated with metabolic pathways such as photorespiration and fatty acid β-oxidation (Del Rio and Lopez-Huertas, 2016). Over-accumulation of ROS can damage cellular components, while at low concentration it acts as the signal transduction messenger to regulate developmental processes and stress responses (Sandalio and Romero-Puertas, 2015; Del Rio and Lopez-Huertas, 2016; Kohli et al., 2017). It was proposed that the potential toxicity and the energetic costs associated with their detoxification determine the positive or negative roles of ROS during the abiotic stress response (Choudhury et al., 2017), and upon ROS accumulation exceeding a certain level ROS toxicity could not be prevented by its scavenging system causing, oxidative damage to plant macromolecules and cell structures, leading to the inhibition of plant growth and development (Hossain et al., 2015). In some cases, ROS contributes to the plant stress tolerance by activating the acclimation pathways, and exogenous application of ROS reinforces plant abiotic stress resistance (Hossain et al., 2015).

Peroxisomes which exist in nearly all the eukaryotic cells are one of the major sources of cellular ROS production (Del Rio and Lopez-Huertas, 2016). They are highly dynamic metabolically active organelles that participate in different cellular processes including development, morphogenesis and response to stresses (Hu et al., 2012). It has been reported that peroxisomes could rapidly sense environmental changes and modify their metabolism and dynamics accordingly through the regulation of ROS (Sandalio and Romero-Puertas, 2015; Del Rio and Lopez-Huertas, 2016). In addition, it has been demonstrated that peroxisomes contribute to the ROS-mediated senescence (Giordano and Terlecky, 2012; Jajic et al., 2015). Peroxisomes could also affect transcription and/or translation by altering the function of key regulatory proteins via ROS-derived redox modifications (Foyer and Noctor, 2013).

To date, much progress has been made in deciphering the molecular mechanisms that control abiotic stress signaling pathways (Zhu, 2016; Kohli et al., 2017; Pareek et al., 2017; Kollist et al., 2018). However, the full details of the network are far from clear with large gaps still existing in our understanding of the regulatory networks that control plant response to the adverse environment, especially with regards to the nature and function of plants rapid response to stress (Kollist et al., 2018). It is proposed that by studying the rapid local and systemic responses to stress, novel pathways that may potentially be used in improving the tolerance of plants to stresses can be identified (Zhu, 2016; Kollist et al., 2018). Characterization of the previously functionally unknown genes that were involved in this process will be important for enhancing our knowledge of plants gene regulatory networks that operate in response to stress (Niehaus et al., 2015). In Arabidopsis in 2015, nearly half of all Arabidopsis genes were completely functionally unknown or poorly characterized (Niehaus et al., 2015). These genes could potentially involve critical signaling pathways that have not yet been identified. Numerous attempts to uncover the biological role of proteins of unknown functions, enumerating their functional significance in growth, development, survival, and response to adverse environmental conditions have been reported in a diverse range of organisms (Horan et al., 2008; Luhua et al., 2013; Dhanyalakshmi et al., 2016).

To identify novel proteins that rapidly respond to salt tolerance in Arabidopsis, we analyzed the stress related transcriptome profiling and searched for genes that rapidly respond to exogenous abiotic stress. *Salt UP-regulated* gene A (*SUPA*) was a candidate for further characterization in this study. We showed that *SUPA* rapidly responds to abiotic stress at the transcriptional level, while localized in the peroxisome. Overexpression of *SUPA* gene causes strong morphological alternations and plant tolerance to abiotic stresses in Arabidopsis and poplar. Our results expand our understanding of the rapid responses to abiotic stress in plants.

## Materials and Methods

### Plant Materials and Growth Conditions

Arabidopsis (*Arabidopsis thaliana* cv Columbia) seeds were sterilized and placed on AM + medium [solid half strength Murashige and Skoog (MS) medium (Murashige and Skoog, 1962] with 0.5 g/l MES, 10 g/l Sucrose and 0.8% agar), and stratified for 2–4 days at 4°C before being placed in the growth chamber (22°C, 16 h light/8 h darkness 100 μmol m^–2^ s^–1^, and a relative humidity of 70%).

To generate *SUPA*-overexpression lines, *SUPA* genomic DNA fragment (At5G65300) was generated by PCR using the primers (F: 5′-GGGGACAAGTT TGTACAAAAAAGC AGGCTTAATGGAATGCAGAAAACAC-3′) and (R: 5′- GGG GACCACTTTGTACAAGAAAGCTGGGTATTAATAAACTCG TTGCCG-3′), then sub-cloned into the PDONR207-vector for sequence confirmation. The genomic DNA fragment of *SUPA* was inserted into the pK7WG2 vector via gateway technology (Karimi et al., 2002). To construct the *SUPA* promoter: GUS line, the 5′-flanking DNA of the *SUPA* coding region was amplified with *SUPA*-specific primers (F: 5′-GGGGACAAGTTTGT AC AAAAAAGCAGGCTTACATCTTTTATCAACACAATCAC TTT-3′; and R: 5′- GGGGACCACTTTGTACAAGAAAGCTGG GTATTTTCTATCTTCTTCGTTCTCTTTT -3′). The 1,526 bp PCR fragment was cloned into the pBGWFS7 vector for sequence confirmation. The coding sequence (CDS) of *SUPA* was also infused with GFP at the N- or C- terminal by using pK7WGF2 and pK7FWG2 vectors via gateway technology, respectively (Karimi et al., 2002). The CDS of *SUPA* was inserted into the pEarlyGate301 vector via gateway technology to generate the transgenic lines expressing *SUPA-HA* infusion construct (Karimi et al., 2002). All the constructions were transformed into Arabidopsis Col-0 by Agrobacterium mediated transformation as described in Clough and Bent, 2010. After selection and molecular verification, we obtained a total of 7 independent *SUPA*-overexpressing lines, 8 *SUPA* promoter: GUS lines, 5 *GFP-SUPA* expressing lines, 6 *SUPA-GFP* expressing lines and 8 *SUPA-HA* expressing lines.

The *supa* mutant (SALK_069313) was obtained from the Arabidopsis stock center (TAIR). The identification of a homozygous mutant was performed as described previously (Brown et al., 2005), and the primers used in this analysis are listed in Supplementary Table 12.

For ectopic expression of *SUPA* in poplar, Shanxin yang (*P. davidiana* × *P. bolleana*) transformation was performed as previously described (Wang et al., 2011). The *35S: SUPA* vector generated above was used for transformation, as well as obtained 10 *SUPA*-overexpression poplar lines.

### GUS Straining

Histochemical staining for GUS activity in transgenic plants and various issues was performed as the protocol described in Jefferson et al. (1987).

### Plant Stress Treatment

The Arabidopsis *SUPA* expression analysis in response to ABA, NaCl, cold or osmotic stresses, treatments were performed as described in Zhu et al. (2010). Briefly, 7 days old seedlings were immersed in 1 μM ABA, 150 mM NaCl, 300 mM mannitol or transferred to 4°C, and the materials were harvested for RNA extraction at the time indicated. All the experiments were repeated independently at least three times.

For the Arabidopsis seed germination assay in stress conditions, approximately 100 seeds each for the wild type and *SUPA* overexpression lines were planted on AM + medium supplemented with various concentrations of ABA or NaCl, respectively, and were kept at 4°C for 2 days before being moved to the growth chamber. Germination (emergence of radicles) was scored daily for 10 days. The vertical germination and growth assays were performed in a similar manner, except that the plates were placed vertically on a rack. Plant growth was monitored and photographed after 7 days. All the experiments were repeated at least three times.

For preparation the samples that were used to measure the concentration of H_2_O_2_ and the level of total anti-oxidative capacity (T-AOC), 7 days old Col-0 and *SUPA* overexpression lines seedlings were treated with different concentration of NaCl (100 mM NaCl or 150 mM NaCl) and ABA (0.5 μM ABA and 1 μM ABA) for 3 h, and the seedlings incubated with water were used as control. After treatments, all samples were immediately frozen in liquid nitrogen and stored at −80°C for further experiments.

For Arabidopsis growth assays in different light conditions, seeds grown on the AM+ plates were placed in white light, blue light, far red light or dark for growth. Plant growth was monitored and photographed after 7 days. All experiments were repeated three times independently, and the average means were calculated.

For the poplar NaCl tolerance assay, the regenerated plantlets were grown on 0.1 mg/L naphthylacetic acid (NAA) containing MS medium with various concentrations of NaCl (50, 100, and 150 mM, respectively), and pictures were taken 21 days after treatment. For adult poplar plant NaCl treatments, 1-month-old poplars grown in soil were watered with 150 mM NaCl every 2 days. For drought treatments, water was withdrawn from 1-month-old seedlings grown in the soil. Plant growth was monitored and pictures were taken at day 32 for drought and salt stress treatments. All experiments were repeated three times independently, and the average data was calculated.

For preparation the samples that used for measuring the concentration of H_2_O_2_ and the level of T-AOC in Populus, wild type and *SUPA* overexpression lines were grown on MS medium with 0.1 mg/L naphthylacetic acid (NAA) for 3-weeks. All experiments were repeated three times independently, and the average data was calculated.

### RNA-Seq Analysis

Total RNA from wild type Col-0 and two *SUPA*-overexpression lines were purified using the Plant RNA Kit (OMEGA; R6827-01) according to the manufacturer’s instructions. 10 μg total RNA was used for RNA-Seq analysis using the BGISEQ-500 platform (BGI, China) with paired-end sequencing, and 3 independent biological repeats were performed. In total, 9 strand-specific RNA-Seq libraries were sequenced in this study using the dUTP method (Parkhomchuk et al., 2009). The paired-end reads were aligned to the *Arabidopsis thaliana* genome using tophat-2.0.11 with an anchor length of more than 8nt for spliced alignments (Kim, 2013). Only reads that could be uniquely aligned were retained for subsequent analysis. The expression levels of each gene were normalized as fragments per kilobase of transcript per million mapped reads (FPKM) (Trapnell et al., 2010). A cutoff with expression fold change > 2 and *p* < 0.05 was used for identifying the differentially expressed genes. The data were deposited in NCBI Sequence Read Archive (SRA)^1^ with accession number PRJNA634204. To verify the RNA-Seq results, qPCR was used as previously described (Zhu et al., 2012). The primers used for qPCR are listed in the Supplementary Table 12.

### RNA Extraction, Reverse Transcriptase-PCR and Quantitative Real-Time PCR Analyses

Total RNA was extracted using the Plant RNA Kit (OMEGA), as instructed by the manufacture. RNA concentration and purity were determined using NANODROP ONE (Thermo Fisher Scientific, United States). For quantitative real-time PCR, materials were harvested and the cDNA was synthesized with the Prime Script^TM^ RT reagent Kit with gDNA Eraser (TaKaRa, Japan) and qPCR was performed with TB Green^TM^ Premix Ex Taq^TM^ II (TaKaRa). The gene specific primers are listed in Supplementary Table 12. The relative mRNA was determined by normalizing the PCR threshold cycle number with Actin, which optimized in the previous study (Czechowski et al., 2005). All RT-qPCR was conducted in triplicate biological replications and subjected to statistical analysis. The delta-delta Ct method was used for analyzing the relative gene expression as described previously (Yuan et al., 2006). At least three independent biological repeats were performed for each data set. Data represent mean values and standard errors (SE).

### Subcellular Localization of SUPA Protein

Arabidopsis expressing *SUPA-HA* were crossed with the peroxisome marker PX-YK Arabidopsis line (Nelson et al., 2007), and the F1 progeny were used for immunostaining using the protocol from Sauer et al. (2006) with minor modifications. Briefly, 4-day old seedlings were fixed in solution containing 4% paraformaldehyde (Sigma, cat. no. P6148) in 1 × PBS supplemented with 0.1% Triton X-100 (Carl Roth, cat. no. 3051.2) in vacuum desiccator for 60 min at room temperature, and then washed two times for 30 min with 1 × PBS at room temperature. After chlorophyll was removed, the samples were incubated with 2% Driselase (Sigma, cat. no. D9515), and 3% IGEPAL CA-630 (Sigma, cat. no. I3021) subsequently, and washed with 1 × PBS for 30-60 min after each incubation. The materials were immersed in 3% BSA for 60 min at room temperature. The primary antibody solution (usually 100–150 μL) was then added to the 3% BSA solution and incubated at 37°C for at least 240 min or overnight at 4°C. The samples were washed two times for 30 min with 1 × PBS at room temperature. The secondary antibody (1:2,000) was added and incubated with the sample for 3 h at 37°C. The samples were harvested after being washed for 30 min with 1 × PBS at room temperature, and then observed using a Zeiss, LSM880 microscope. The antibodies used in this study include rabbit anti-HA (1:1,000; Gene Tex; GTX73046), mouse anti-GFP (1:1,000; TRANS; HT801-01), mouse anti-RFP (1:1,000; Eno Gene; E12-011), Goat anti-Mouse IgG (H + L) Cross-Adsorbed Secondary Antibody, Alexa Fluor 546 (Thermo Fisher Scientific; A11008) and Goat anti-Rabbit IgG (H + L) Cross-Adsorbed Secondary Antibody, Alexa Fluor 488 (Thermo Fisher Scientific; A11003). Pictures were taken using a Zeiss LSM 880 confocal scanning microscopes with Airyscan (Huff, 2016).

### ROS Staining and Quantitative Measurements

DAB and NBT staining were used to detect different ROS based on the protocol of Zhu et al. (2013). Briefly, Arabidopsis or poplar leaves at the same stage and positions were submerged into 1 mg/mL DAB or 2 mg/mL NBT solution for 4 h in the dark, and then observed under the microscope after bleaching. Hydrogen peroxide was determined using the Micro Hydrogen Peroxide (H_2_O_2_) Assay Kit (Solarbio, China) as described by the manufacturer. T-AOC was determined using the Micro Total Antioxidant Capacity (T-AOC) Assay Kit (Solarbio, China) as described by the manufacturer.

## Results

### *SUPA* Is Rapidly Induced by ABA and Abiotic Stress Conditions

Previously, a systematic study on the transcriptional response to high salinity of differential cell layers and developmental stages of Arabidopsis was performed (Dinneny et al., 2008). To identify the novel stress-responsive genes, firstly we queried their transcriptomic dataset, and focused on the genes that previously uncharacterized but rapidly respond to salt stress (< 30 min). Secondly, the selected genes were further analyzed in the public transcriptomic database Genevestigator (Hruz et al., 2008) to check their expression changes in response to other stresses and stress related phytohormones, such as abscisic acid (ABA), salicylic acid (SA),indole-3-acetic acid (IAA), *P. syringae* and drought. At5g65300 (*SUPA*), which is one of the candidates that rapidly and strongly respond to those stresses, is further functionally characterized in this study.

**FIGURE 1:**
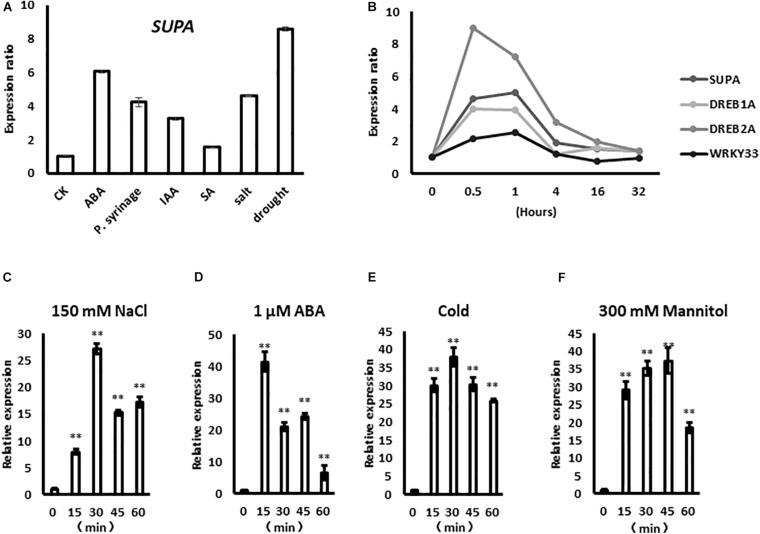
*SUPA* responds rapidly to abiotic stresses. **(A)** *SUPA* transcript is induced by various stress factors based on the data from Genevestigator. For ABA treatment, 2-week old Arabidopsis Col-0 were treated with 50 μM ABA for 90 min; for P. syringae treatment, leaves of 4-week old Col-0 plants with 10e5 cfu*cm^–2^ bacteria for 24 h; for IAA treatment, 7-day old Col-0 seedlings with 1 μM for 1 h; for SA treatment, leaves from 3-week old Col-0 grown on soil were treated with 1 mM SA for 6 h; for salt treatment, 16-day old Col-0 seedlings were treated with 150 mM NaCl for 3 h; for drought treatment, 2-week old Col-0 seedlings were exposed to a stream of air in a clean bench for 4 h; after treatments, RNA were extracted and used for gene expression analysis. The control was set as 1, and the expression levels of each treatments were calculated compared with the gene expressions of their untreated samples, respectively. **(B)** The expression level of *SUPA* in response to NaCl at different time points based on the public stress-related transcriptomic data. The expression data of some well-characterized salt responsive factors, such as *DREB1A*, *DREB2A*, and *WRKY33* were also shown as positive controls. In this report, seedlings were planted on AM + medium for 5 days, then were transferred to AM + medium with 140 mM NaCl for 0, 30 min, 1, 4, 16, and 32 h, respectively. **(C–F)** qRT-PCR analysis of *SUPA* expression pattern in response to ABA and various abiotic stresses. Seven-days-old seedlings grown on AM + medium were treated with 150 mM NaCl **(C)**, 1 μM ABA **(D)**, 4°C **(E)**, and 300 mM Mannitol **(F)**. Samples were collected at the indicated time after the initiation of the treatment. Total RNA was extracted from whole seedlings, and real-time PCR analyses were performed with *SUPA* gene specific primers. The relative expression was calculated by using *Actin* as an internal reference. The unstressed expression level was assigned a value of 1. Data represents the average of three independent experiments ± SE. **P < 0.01.

The expression of *SUPA* gene is rapidly up-regulated by several biotic and abiotic factors, such as abscisic acid (ABA), *P. syringae*, indole-3-acetic acid (IAA), salicylic acid (SA), salt and drought based on the data from Genevestigator (Figure 1A), indicating potential roles in plant stress responses. Based on the public large-scale transcriptomic data (Kreps et al., 2002; Kilian et al., 2007; Dinneny et al., 2008), the *SUPA* gene rapidly responds to salt treatment within 30 min, and this expression pattern was similar to the already characterized rapid stress-response transcription factors *DREB1A*, *DREB2A*, and *WRKY33* (Figure 1B). To confirm the results above, we treated the samples under various stress over time, then performed qPCR analysis. Our results showed that *SUPA* transcripts could be up-regulated within 15 min of salt treatment, reached a maximum at 30 min, and then gradually reduced (Figure 1C). After ABA treatment *SUPA* transcripts increased to a maximum level within 15 min and then decreased (Figure 1D). The accumulation of *SUPA* transcripts increased 15 min after cold treatment, and remained at a high level for 60 min (Figure 1E). Under osmotic treatment, *SUPA* transcripts rapidly accumulated to a maximal level within 15 min, and a peak in accumulation was observed at 45 min, with levels subsequently reducing (Figure 1F). These results showed clearly that *SUPA* gene could be rapidly and transiently activated at the transcriptional level by ABA, salt, osmotic and cold stress treatments, indicating it may be involved in plant response to abiotic stresses.

### SUPA Proteins and Its Homologs in Arabidopsis

SUPA protein is composed of 150 amino acids, and at present it was classified as a functionally unknown protein (Data from TAIR). Three of the phylogenetically closest genes were identified through a BLAST search of the full amino acid sequence of SUPA against the complete Arabidopsis genome database, but they showed low similarities (identity < 30%) (Supplementary Figure 1A). Further alignment analysis showed that the three proteins contain several conserved domains, including a “CkKH^∗^kHrqspGvCslCL^∗^ekLSrl” domain at the N-terminal (domain I), serine-rich domain in the middle (domain II), and lysine-rich domain at C-terminal (domain III) (Supplementary Figure 1C). We performed a further search using the above conserved domains as the query, and no additional proteins containing such domains in Arabidopsis genome were identified. More importantly, all three SUPA homologs could also be induced by salt, ABA, cold and osmotic stress within 15 min, suggesting their potential roles in plant stress-response (Supplementary Figure 2). We also searched the *SUPA* homologous in other plant species such as poplar. By using the full amino acid sequence of Arabidopsis SUPA against the complete poplar genome database^2^ with BLAST tools, we found that the gene with the highest identity as Arabidopsis SUPA protein shared more than 50% similarity (Supplementary Figure 1B), moreover, POPTR_0005s07300.1 is Highly homologous with SUPA in Arabidopsis. and we do find the typical conserved domain as in Arabidopsis (Supplementary Figure 1D). Further experimental work is required to check these genes have similar functions.

### *SUPA* Gene Is Mainly Expressed in the Above-Ground Tissues

**FIGURE 2:**
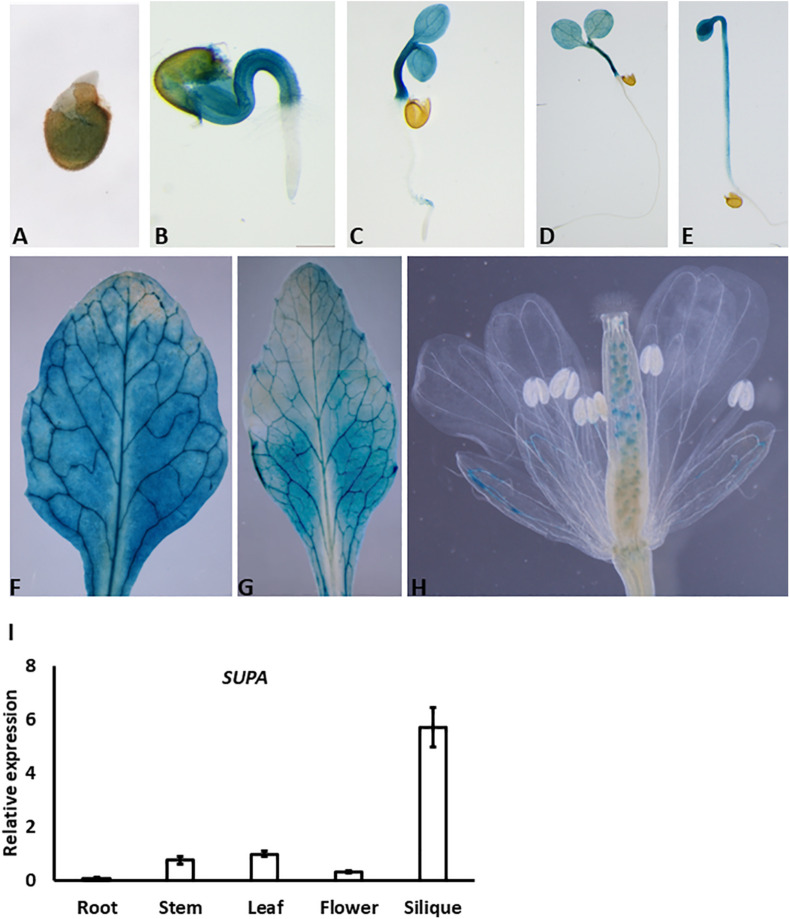
Expression patterns of *SUPA* gene. **(A–H)** *SUPA* promoter activity analyses by GUS staining. Transgenic plants expressing GUS driven by 1.5 kb *SUPA* promoter were constructed, and the GUS activity was analyzed and measured in 1-day old seedlings **(A)**, 2-day seedlings **(B)**, 3-day old seedlings **(C)**, 5-day old seedlings grown in light **(D)** and in darkness **(E)**, cauline leaves of 1-month old plant **(F)**, rosette leaves of 1-month old plant **(G)**, flowers **(H)**. **(I)** qRT-PCR analyses of *SUPA* transcripts in different organs of Arabidopsis plants. Total RNA was isolated from various tissues (root, stem, rosette leaf, flower, and silique) of 5-week-old Arabidopsis (ecotype Col-0). qRT-PCR was performed with *SUPA*-specific primers. Expression of *Actin* was analyzed as the control. *SUPA* expression level in the stem was assigned a value of 1. The data shown represents mean values and standard errors obtained from three independent experiments.

To determine the possible function of the *SUPA* gene, we first examined its expression patterns in various tissues and different developmental stages. We generated *SUPA-promoter: GUS* transgenic plants and SUPA was analyzed using GUS staining. The positive signal was detected at a very early stage (1-day after germination) (Figure 2A), and became strong mainly in the hypocotyl and cotyledon of the 2-, 3-, and 5-days-old seedlings, but not in the root (Figures 2B–D). Interestingly, we did not detect the GUS activity in the root in both light (Figure 2D) and dark conditions (Figure 2E). A GUS signal was also detected in the rosette leaves and cauline leaves of 1-month old plant (Figures 2F,G). In the flowers, expression was detected in the sepal and ovules (Figure 2H). Further qRT-PCR confirmed that the high expression of SUPA exist in stem, leaf, flower and siliques while quite low expression levels in roots (Figure 2I), indicating SUPA may mainly play its function in the above-root tissues.

### Overexpression of *SUPA* Leads to Various Morphological Alterations in Arabidopsis Under Normal Conditions

**FIGURE 3:**
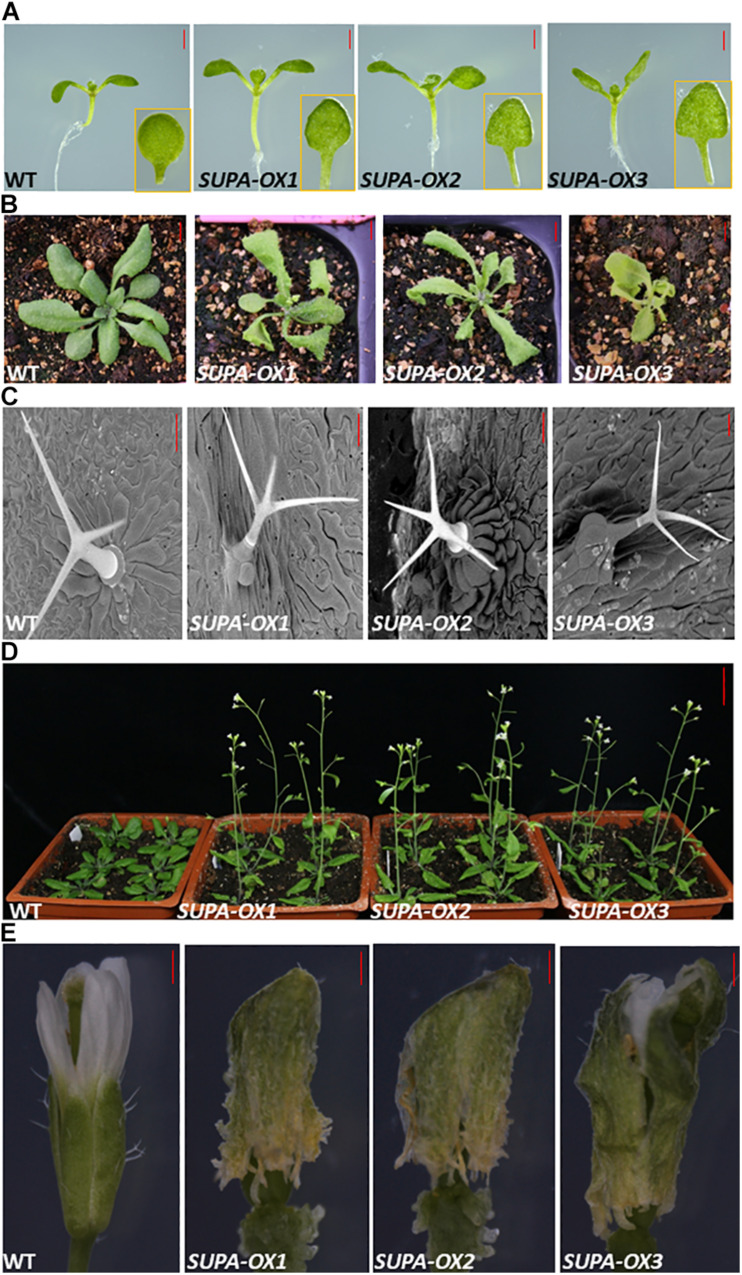
Overexpression of *SUPA* leads to various morphological alterations in Arabidopsis. **(A)** *SUPA-OX* lines have longer hypocotyls and petioles compared to wild type under white light. Photographs were taken from 7 days old seedlings, and the representative pictures are shown. Bar = 1 mm. **(B)** Phenotypes of the soil-grown plants. Photographs were taken from 3-week old seedlings grown in the soil, and the representative pictures are shown. Bar = 5 mm. **(C)** Trichrome phenotypes. Photographs were taken with a SEM, and the representative pictures are shown. Bar = 100 μm. **(D)** Effects of *SUPA* overexpression on Arabidopsis flowering time. *35S: SUPA* transgenic lines flower earlier than wild type. Pictures were taken 5-weeks after sowing. Bar = 2.5 cm. **(E)** Effects of *SUPA* overexpression on flower opening. Photographs from the flowers of 4-week old plants are shown. Bar = 25 μm.

To investigate the function of *SUPA*, we generated transgenic Arabidopsis overexpressing *SUPA*, and observed their growth under normal growth conditions. At the seedling stage, the overexpression lines showed much longer hypocotyl, more curved cotyledons and longer cotyledon petioles compared with wild type under white light (Figure 3A and Supplementary Figures 3A–C). Those phenotypic changes also exist under the far-red light (Supplementary Figures 3A,D,E) and more obvious under the blue light (Supplementary Figures 3A,F,G), while these phenotypic changes disappear in the dark (Supplementary Figures 3A,H). These results showed *SUPA* plays a role in plant growth and development in a light-dependent manner at the seedling stage.

Transgenic Arabidopsis overexpressing *SUPA* grown in the soil at the 3-week-old stage, had curled leaves comparison to wild type (Figure 3B). Additional data from the scanning electron microscope (SEM) showed that the basal cells below the trichrome protruded from the epidermal cells and have distinctive shapes compared to the regular epidermal cells (Figure 3C).

We also observed that overexpression of *SUPA* leads to the early flowering phenotype in Arabidopsis. All 15 transgenic lines generated produced visible inflorescences approximately 7–14 days earlier than wild type (Figure 3D). The flower structures of the transgenic lines do not change compared to wild type. However, the sepal surface of the *SUPA-OX* lines was not as smooth as wild type (Supplementary Figure 4A). At the age of 15 days, the sepal of transgenic lines was not able to open as in wild type and therefore the petal, the carpel, the pistil and the anther remained enclosed inside (Figure 3E and Supplementary Figure 4B). Results from SEM at stage 15 showed that wild type flowers were open at this stage whereas in the overexpression lines the upper parts of the sepal remained closed. (Supplementary Figure 4C), and the basal parts of their sepals were ruptured (Supplementary Figure 4D), which lead to the deformed and twisted petals (Supplementary Figure 4B). The papilla cells of the stigma are much shorter in *SUPA-OX* lines compared to wild type (Supplementary Figure 4E). More importantly, most anthers of the *SUPA-OX* lines did not contact the stigma and this is thought to be due to physical prevention from the enclosed sepal which resulted in most pollen being spread within the carpel instead of the stigma as normally occurs. This lead poor fertilization in transgenic lines. Interestingly, fertilization was successfully restored after manual self-pollination, indicating that the flower has a normal physiological function while anatomical constraints due to the sepal being enclosed inhibits fertilization (Supplementary Figures 4F,G). Our results clearly showed that overexpression of *SUPA* leads to broad phenotypic changes in Arabidopsis under normal growth conditions, indicating that the expression of *SUPA* needs to be tightly controlled to maintain normal growth and development.

The T-DNA insertion line for *SUPA* gene was obtained from the Arabidopsis Biological Resource Center (ABRC), and genotyping results revealed the T-DNA fragment was inserted in the exon of the gene, leading to the termination of *SUPA* protein translation (Supplementary Figures 5A,B). We performed phenotypic analysis, and we did not find any changes between wild type and *supa* mutant both under normal and stress conditions (Supplementary Figure 5C). These results suggested that SUPA together with other 3 proteins may have functional redundancy, possibly explaining why the *supa* single mutant does not generate any phenotypes. In the future, further detailed investigations including generation of double and multiple mutants will help to revile the function of this gene cluster.

### SUPA Protein Is Localized in the Peroxisome

To determine SUPA subcellular localization in native Arabidopsis cells, we firstly fused the CDS of SUPA with GFP at the N- or C- terminal, to generate stable transgenic lines. We found that the SUPA-GFP transgenic lines have more twisted root structures, and do not showed the complete same phenotype compared with the *SUPA*-overexpressing lines (Supplementary Figures 6A,B), suggesting that SUPA-GFP fusion protein may not cause exactly the same effects as SUPA protein alone. For GFP-SUPA, we observed that this line gave no fluorescence under confocal microscope. Therefore, both SUPA-GFP and GFP-SUPA lines could not be used for determining its subcellular localization.

We speculate that the SUPA protein structure maybe disrupted when fused to GFP given that SUPA is a small protein (16.4 kDa) compared to GFP (26.9 kDa). To solve this issue of size we fused the *SUPA* gene with an HA tag and generated transgenic lines. We observed that the phenotypes of *SUPA-HA* transgenic lines were comparable to the *SUPA* transgenic line (Supplementary Figures 6A,B), indicating SUPA-HA fusion protein is expressed and functioned in a manner comparable to the SUPA protein alone.

**FIGURE 4:**
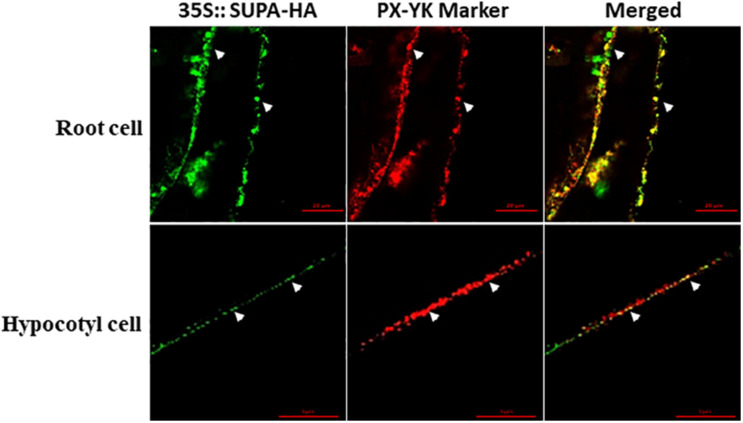
SUPA protein is localized in peroxisomes. Arabidopsis expressing the *SUPA-HA* construct was crossed with the peroxisome marker PX-YK expressing Arabidopsis line. The root cell (up panel) and the hypocotyl cell (low panel) of the 7-day old seedlings from the F1 progeny were used for immunostaining with anti-HA (green) and anti-GFP (red) antibody. Pictures were taken using a Zeiss LSM 880 confocal scanning microscopes with Airyscan. In the up panel, Bar = 20 μm, and in the low panel, Bar = 5 μm.

To reveal the subcellular localization of SUPA protein, we crossed *SUPA-HA* overexpression lines with several well-characterized cell organelle marker lines, including 35s:GFP-8a for Lysosome, Mt-RK for mitochondria, and PX-YK for peroxisomes (Nelson et al., 2007). The F1 progeny were used for immunolocalization studies using HA- and GFP/RFP- specific antibodies (Sauer et al., 2006). Our results showed that the HA signal exclusively co-localized with the signal from the peroxisome marker PX-YK, both in the cells of root and hypocotyl (Figure 4), but not co-localized with the signal of Lysosome marker 35s:GFP-8a and mitochondria marker Mt-RK (Supplementary Figure 7). These result indicated that SUPA protein mainly localized to the peroxisomes of plant cells.

### Overexpression of *SUPA* Leads to the Accumulation of ROS

**FIGURE 5:**
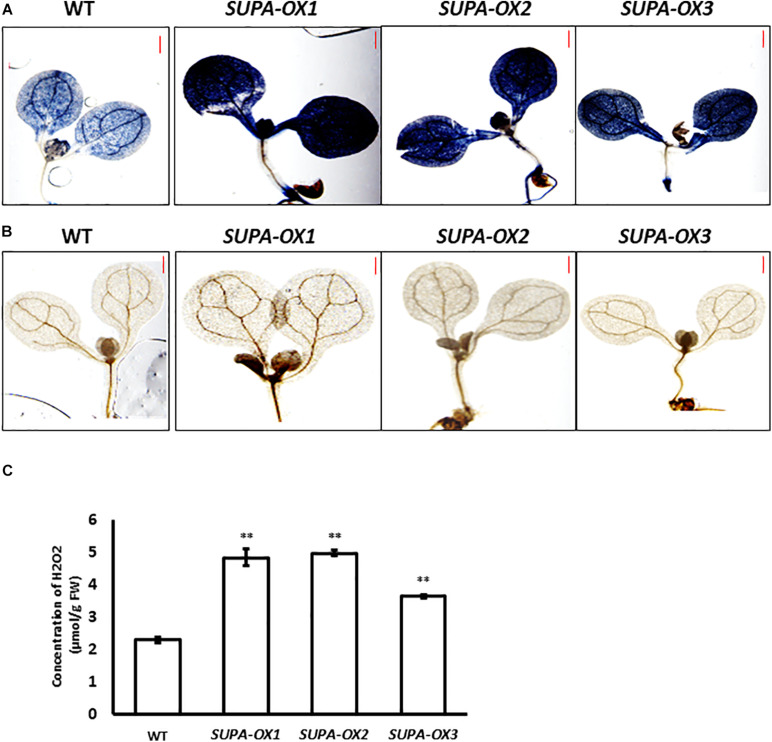
Overexpression of *SUPA* leads to enhanced ROS levels. **(A)** NBT staining for superoxide in WT and *SUPA-OX* lines. 7-day old seedlings were used for staining. Bar = 0.5 mm. **(B)** DAB staining for hydrogen peroxide in WT and *SUPA-OX* lines. 7-day old seedlings were used for staining. Bar = 0.5 mm. **(C)** Quantitative measurements of ROS content in WT and *SUPA-OX* lines. Data represent the mean ± SE of three independent measurements. **p < 0.01.

Since SUPA protein is localized in the peroxisomes that are important sites for ROS production, we predicted that ROS levels may be altered in the transgenic lines. To test this hypothesis, we stained the seedlings of *SUPA*-overexpression lines and wild type with diaminobenzidine (DAB, for H_2_O_2_) and nitroblue tetrazolium (NBT, for superoxide) which indicate the presence of ROS. The results showed that the transgenic lines had much stronger signals with both stains compared to wild type (Figures 5A,B). The following quantitative measurements of the H_2_O_2_ levels corroborated these observations (Figure 5C). These results showed that *SUPA* overexpression leads to the ROS over-accumulation in the transgenic Arabidopsis under normal growth conditions.

### Heat Shock Proteins Had Enhanced Expression at the Transcription Level

**FIGURE 6:**
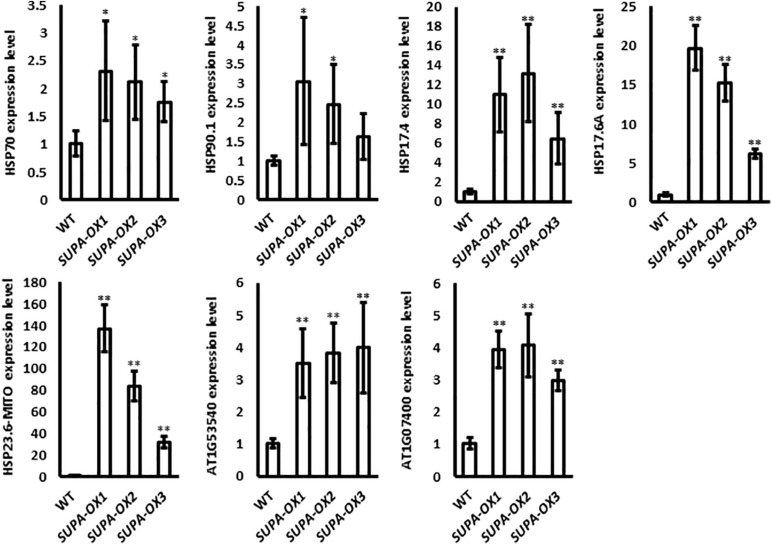
Expression of heat shock protein family genes in wild type and *SUPA*-overexpression lines. 7-day old seedlings grown in normal conditions were used in this experiment. *HSP*-gene specific primers were used and the *Actin* gene specific primers were used as the control. The gene expression levels in WT were set as 1. Data represents the average of three independent experiments ± SE. *p < 0.05. **P < 0.01.

ROS has emerged as major regulatory molecules in plants (Waszczak et al., 2018), and can effectively induce the heat shock proteins (HSP), which belong to one of the major transcription factor families that respond to various environmental stresses (Zhu, 2016; Jacob et al., 2017). Our results showed that overexpression of *SUPA* leads to the accumulation of ROS (Figure 5). We further checked the expressions of genes coding for the heat shock proteins. Real-time qPCR results showed that all the HSPs we checked, including *Hsp70*, *Hsp90.1*, *Hsp17.4*, *Hsp17.6A*, *Hsp23.6-M*, *Hsp20*-like protein (*At1g53540* and *At1g07400*), have enhanced transcript levels in the *SUPA* overexpression lines compared with wild type (Figure 6), which is probably due to the increased ROS levels.

### Overexpression of the *SUPA* Gene Changed the Plant Response to Abiotic Stresses

**FIGURE 7:**
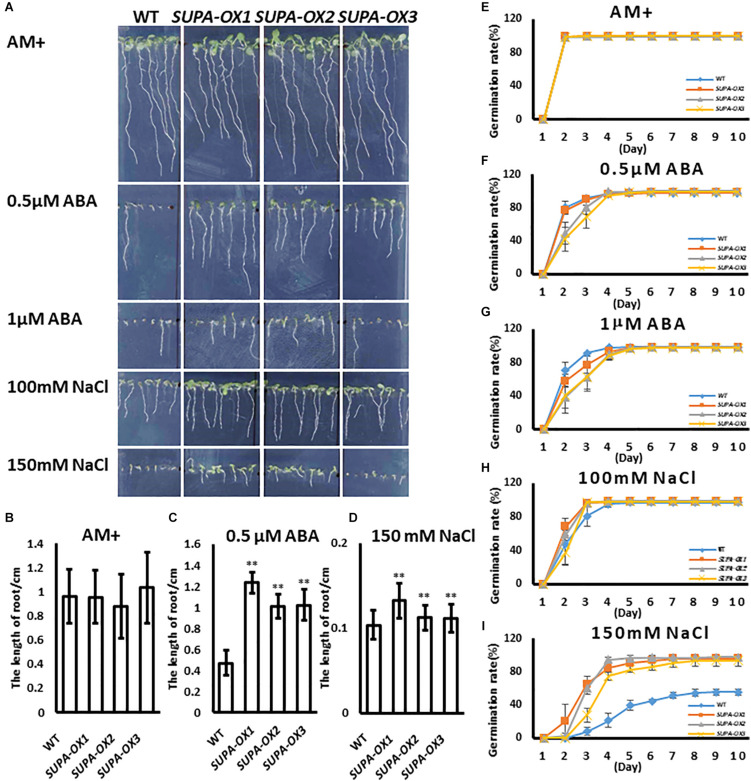
Overexpression of *SUPA* enhances Arabidopsis response to abiotic stresses at seedling stage. **(A)** *SUPA*-overexpression lines were insensitive to ABA and salt stress. Wild type and three *SUPA*-overexpression lines were grown vertically on AM + medium with or without ABA or NaCl, and photos were taken on day 7 after stratification. Representative images from the three independent experiments are shown. **(B–D)** Statistically analysis of root length. The root length of seedlings grown with or without **(B)** ABA **(C)** and NaCl **(D)** were measured using image J and statistically analyzed. Results are presented as means and standard errors from three independent experiments (*n* > 15), *p < 0.05, **p < 0.01. **(E–I)** Effects of ABA and NaCl on seed germination. Wild type and *SUPA* overexpression line seeds were sown on AM + medium **(E)**, or AM + medium containing. 0.5 μM ABA **(F)**, 1 μM ABA **(G)**, 100 mM NaCl **(H)**, 150 mM NaCl **(I)**, Germination (emergence of radicles) was scored daily for 10 days. All results are presented as means and standard errors from three independent experiments (about 100 seeds for each repeat).

ROS play key roles in plant acclimation to abiotic stresses (Choudhury et al., 2017). It was reported that moderate accumulation of ROS within cells or the exogenous application of H_2_O_2_ significantly enhanced plant tolerance to abiotic stresses (Ozaki et al., 2009; Ishibashi et al., 2011; Sun et al., 2016). Since *SUPA* overexpression leads to the over-accumulation of ROS and constitutively induces many heat shock protein family genes, the hypothesis was that transgenic lines may change their response to abiotic stresses. To test this hypothesis, we investigated the seed germination and early seedling growth under various stress conditions in transgenic lines vs. wild type. Under normal growth condition, wild type and transgenic plants had similar germination ratios and displayed healthy growth (Figures 7A,B,E). Regarding ABA treatment, the germination ratios between wild type and transgenic lines are comparable (Figures 7F,G), while the post-growth of the seedlings were different. As shown in Figure 7, the *SUPA-OX* lines were more insensitive to the ABA (Figures 7A,C). Under salt treatment, the germination ratio of *SUPA-OX* lines was higher and post-germination growth was higher than wild type, especially on medium containing 150 mM NaCl (Figures 7A,D,H,I). These results indicated that overexpression of *SUPA* conferred plant tolerance to ABA and salt at the early seedling stage of growth.

### H_2_O_2_ Concentration and Total Antioxidant Capacity (T-AOC) Level Was Altered After Stress Treatment

ROS plays a key role in plant response to abiotic stresses (Choudhury et al., 2017). We measured the H_2_O_2_ levels of wild type and transgenic lines after stress treatment. Our results showed that under normal conditions the ROS levels in transgenic lines were much higher than wildtype (Supplementary Figure 8). However, compared with wildtype, transgenic lines accumulate lower ROS levels after ABA or NaCl treatment, particularly after ABA treatment (Supplementary Figure 8). In addition, the total antioxidant capacity of all transgenic lines was higher than wild type plants in stress conditions (Supplementary Figure 8), which showed that the transgenic lines had a stronger free radical scavenging capability than the wild type plants.

### Overexpression of *SUPA* Leads to the Broad Transcriptomic Changes

**FIGURE 8:**
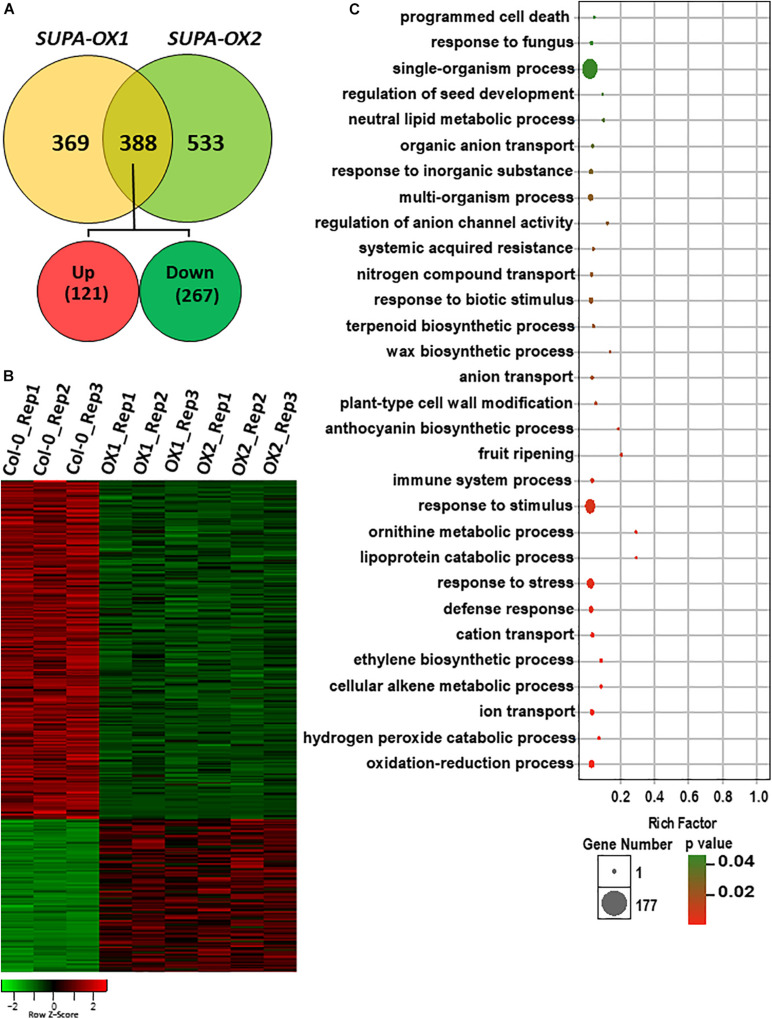
Overexpression of *SUPA* leads to broad transcriptomic changes. **(A)** Venn diagram showing the overlap of DEGs between two *SUPA*-overexpression lines. 388 DEGs were presented in both *SUPA*-overexpression lines, with 121 up-regulated and 267 down-regulated. **(B)** Heat map representation of the differentially expressed genes. Numbers on the nodes display major gene groups based on expression patterns. Color scale shows log2 signal intensity values. **(C)** Go analysis of the differentially expressed genes. The top 30 significant GO terms of differential expressed genes are presented graphically. The *X*-axis represents the value of –log10 (*p*-value). The *Y*-axis indicates the name of the GO term. The size of each point is proportional to the differentially expressed genes associated with the GO terms.

*SUPA* overexpression leads to strong morphological changes under both normal and stress conditions, which is indicative of broad gene expression changes at the molecular level. To test this hypothesis, we performed RNA-Seq analysis using the hypocotyls and cotyledons from wild type and two *SUPA-OX* lines (*SUPA-OX1* and *SUPA-OX2*). The results revealed 757 and 921 differentially expressed genes (DEGs) in two independent *SUPA*-overexpression lines, respectively, using a threshold fold change > 2 and a significance level of *p* < 0.05 (Rosenwasser et al., 2011). Of these DEGs, 388 genes were found to be present in both overexpression lines, with 121 genes being up-regulated (31.2%) and 267 genes being down-regulated (68.8%) (Figures 8A,B and Supplementary Table 1). These 388 genes were regarded as *SUPA*-regulated genes. Gene Ontology (GO) enrichment analysis showed that overrepresented categories were molecular functions related to plant stress response (such as oxidation-reduction process, defense response, defense response and defense response), plant development (such as programmed cell death, ethylene biosynthetic process, fruit ripening, plant-type cell wall modification, wax biosynthetic process and regulation of seed development), and metabolic pathways (such as cellular alkene metabolic process, ornithine metabolic process, lipoprotein catabolic process, regulation of anthocyanin biosynthetic process and neutral lipid metabolic process) (Figure 8C and Supplementary Table 2), which is consistent with the morphological observations. We also noticed that 369 and 533 DEGs were specifically expressed in *SUPA-OX1* and *SUPA-OX2* transgenic line, respectively (Figure 8A). GO analysis indicated that genes specifically expressed in *SUPA-OX1* was enriched in response to mechanical stimulus, oxidative stress, and chitin, lipid binding and localization, and ethylene metabolic pathways (Supplementary Table 3). For DEGs that are specifically expressed in *SUPA-OX2*, enrich categories were mainly related to lipid transport, reduction and localization, as well as oxidative reduction and transitional metal ion transport were significantly enriched (Supplementary Table 4).

Since SUPA is localized in the peroxisome, we analyzed the peroxisomal related genes that differentially expressed in both *SUPA-OX* lines. Of the peroxisomal genes defined previously (Ebeed et al., 2018), a total of 20 genes (7 upregulated and 13 down regulated) were differentially expressed in *SUPA* overexpression (Supplementary Table 5). Gene Ontology (GO) enrichment analysis showed that overrepresented categories related to response to oxidative stress, peroxidase activity, and cofactor binding were significantly enriched (Supplementary Table 6).

In summary, overexpression of *SUPA* leads to broad gene expression changes at the transcriptional level, which may result in the phenotypic changes in the transgenic lines.

### Overexpression of *SUPA* in Poplar Leads to Strong Morphological Alterations

**FIGURE 9:**
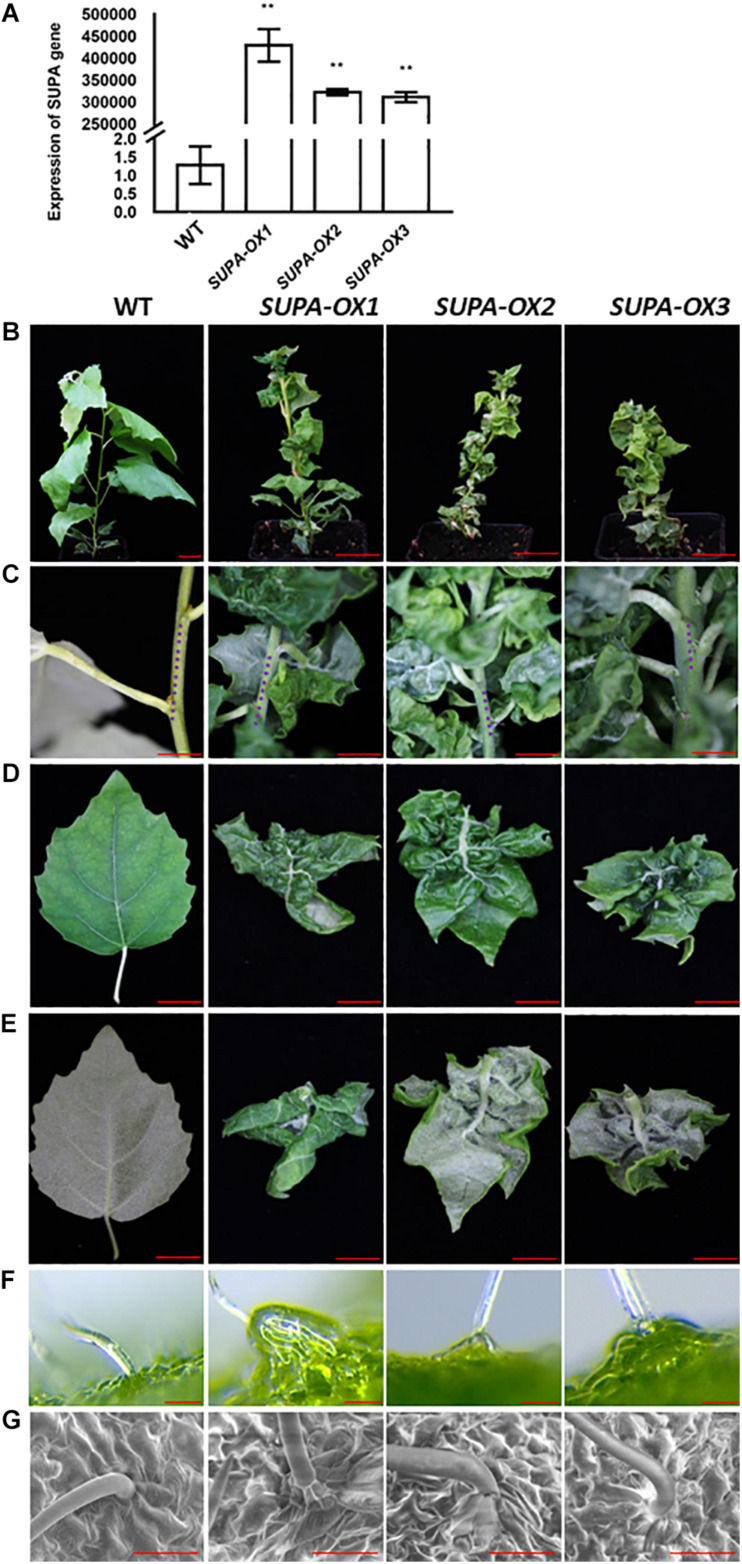
Overexpression of *SUPA* in poplar leads to multiple phenotypic changes under normal growth conditions. **(A)** qRT-PCR verification of the transgenic poplars overexpressing the *SUPA* gene. Leaves from 3-week old plants were harvested and used for RNA extraction, and the qPCR analysis was performed with *SUPA*-gene specific primers. Data represents the average of three independent experiments ± SE. **P < 0.01. **(B–G)** Phenotypic categories of transgenic plants overexpressing *SUPA* genes. 2-month old wild type and transgenic poplars are shown in **(B)** Bar = 4 cm; compared with wild type, the internodes of the *SUPA* overexpression plants are much shorter **(C)** Bar = 2 cm, blue dots indicate the length of the internode; the front side **(D)** Bar = 1.5 cm; back side **(E)** Bar = 1.5 cm and the trichrome observed with Stereo fluorescence microscope (Leica M205FA) **(F)** or by SEM **(G)** of the wild type and transgenic lines are shown. Bar = 50 μm in the **(F,G)**.

Overexpression of *SUPA* changes the leaf shape and increases plant tolerance to abiotic stresses in Arabidopsis, and we further investigated if this gene functions in other plant species such as poplar. We ectopically expressed Arabidopsis *SUPA* gene in poplar, and 10 independent transgenic lines were obtained. We analyzed expression level by q-PCR with leaves, the results showed that *SUPA* expressions in transgenic lines were significantly higher than WT, of which data from 3 representative lines are shown (Figure 9A). The transgenic lines exhibited dwarf phenotypes with severe morphological alterations under normal growth conditions (Figure 9B). All the *SUPA-OX* lines display shorter internodes (Figure 9C), and their leaves are dramatically wrinkled and curved upward (Figures 9D,E), which likely results from the differential development of the right and left sides of the leaf blade. We also observed that the basal cells below the trichrome of transgenic poplar were disformed, and they protruded from the epidermal cells and have distinctive shapes compared to the regular epidermal cells, which is quite similar as we observed in Arabidopsis (Figures 9F,G). Such phenotypes appear from the tissue culture stage, and remain after being transplanted to soil. These results suggested that some conserved signaling pathways may be activated which result in similar phenotypes when Arabidopsis *SUPA* is overexpressed in Arabidopsis and poplar.

### *SUPA* Overexpression Leads to Multiple Abiotic Stresses Alterations in Poplar

**FIGURE 10:**
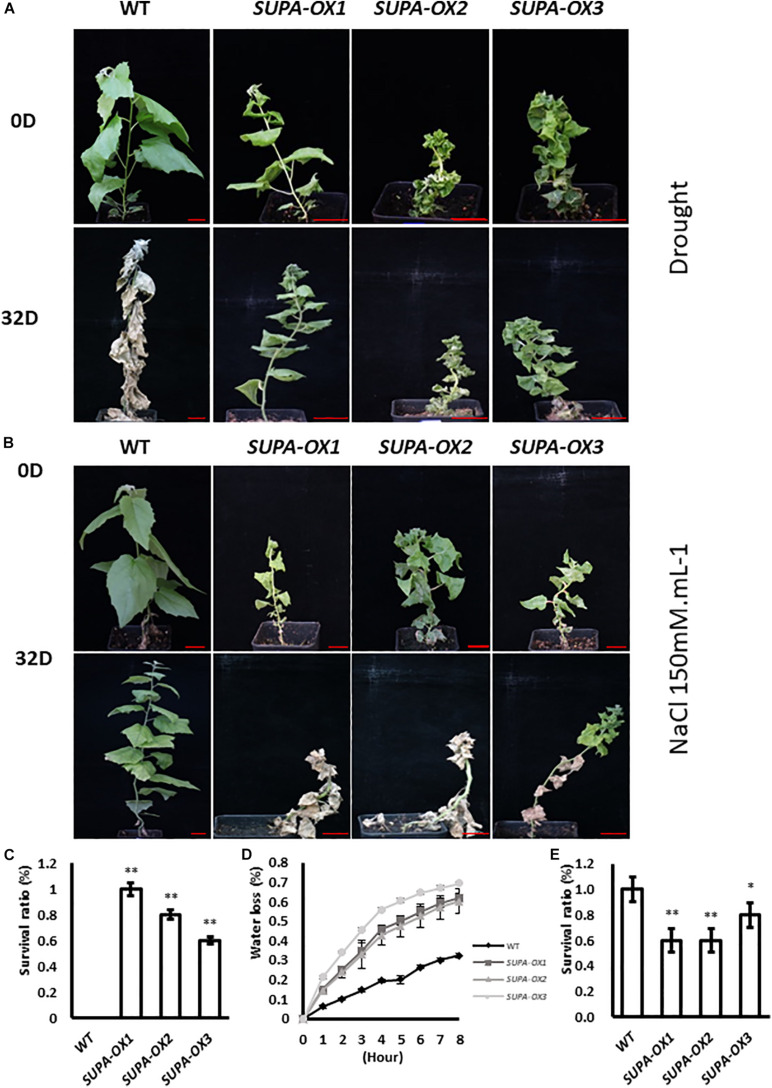
Ectopic expression of *SUPA* gene in poplar alters plant tolerance to salt and drought. **(A,B)** Growth of wild type and three independent transgenic poplars before and after salt or drought treatment. 1-month old poplar plants were treated with water withdrawal **(A)** or 150 mM NaCl **(B)**, pictures were taken before and after 32-day treatment. 4 independent repeats were performed and the representative plants were shown. Bar = 4 cm. **(C–E)** Statistical analysis of the survival between wild type and three independent transgenic lines after salt treatment **(C)** or drought treatment **(E)**, and the water loss rate **(D)**. The water loss rates of cut leaves were measured at the indicated times after their excision. The water loss was calculated as the percentage of initial fresh mass. The results are presented as means and standard errors from three independent experiments. *p < 0.05. **P < 0.01.

Our results showed that enhanced expression of *SUPA* in Arabidopsis leads to the over-accumulation of ROS, and enhances plant tolerance to abiotic stresses (Figure 5 and Supplementary Figure 8). We therefore tested if the ROS levels and stress tolerance properties were changed in transgenic poplar. We first checked the H_2_O_2_ levels of wildtype and transgenic lines grown under normal growth conditions. As in Arabidopsis, higher accumulation of ROS was observed in the *SUPA*-overexpression poplar transgenic lines, revealed by our DAB and NBT staining, as well as H_2_O_2_ measurements (Supplementary Figures 9A,B), and the total antioxidant capacity was also increased (Supplementary Figure 9C), indicating the transgenic poplar may also change their stress tolerance. We further performed drought or salt treatment. For drought treatments, 100% of the *SUPA*-overexpression lines grew well and maintained green coloration, while 95% wild type plants were withed and dead after 32 days of water withdrawal (Figures 10A,C). Interestingly, results from water-loss measurements showed that the water-loss rate from detached leaves were much higher in the transgenic lines (Figure 10D), indicating the drought tolerant phenotype of the *SUPA-OX* lines was not due to reduced water loss from the leaves, and other yet-to-known molecular mechanism may contribute to the increased drought tolerance. Further investigation of the metabolic and molecular changes is required to fully explain these results.

Regarding salt treatment, the overexpression lines remained alive but became withered when grown on 50 or 100 mM NaCl-containing medium at the tissue culture stage, while the wild type plants grow normally (Supplementary Figure 10). For the soil-grown plants, after 32 days treatment with 150 mM NaCl, 50% of the transgenic lines were dead, whereas 100% of wild type plants were alive (Figures 10B,E). These results highlight that unlike the *SUPA*-mediated stress-tolerant phenotypes displayed in Arabidopsis, the overexpression of *SUPA* in poplar dramatically reduced plant tolerance to salt (Figure 10B and Supplementary Figure 10).

## Discussion

Plants have evolved complicated mechanisms to cope with abiotic stresses, however, our knowledge of the molecular mechanisms behind these processes are still limited. In Arabidopsis, still nearly one-half of all Arabidopsis genes are functional unknown, and it is possible and extremely important to identify the novel genes that play important roles in plant stress tolerance.

Peroxisomes are highly versatile and assist plants in adapting to changing environments through cellular metabolic adjustments, such as adjustments in photorespiration and fatty acid ß-oxidation, ureide, and phytohormone (auxin and jasmonic acid) metabolic rates (Hu et al., 2012). Peroxisomes are also the major sites of intracellular ROS production (Del Rio and Lopez-Huertas, 2016), which has both positive and negative effects on plant tolerance to stresses depending on ROS concentration. It was reported that peroxisomes could rapidly respond to stress, and rapidly accumulate ROS and induce downstream stress-related gene expressions (Rodríguez-Serrano et al., 2016). However, the molecular mechanism is unclear. To the best of our knowledge, until now no single peroxisome-localized protein that was able to rapidly respond to stress (within 15 min) and contribute to the accumulation of ROS has been reported.

In this study, we identified a novel peroxisome-localized factor *SUPA* which rapidly responds to ABA and abiotic stresses at the transcriptomic level. Overexpression of *SUPA* in both Arabidopsis and poplar significantly enhanced their ROS levels, and the transgenic lines dramatically changed their phenotypes both under normal growth and stress conditions.

### *SUPA* Is Rapidly Responsive to Abiotic Stresses When Localized in the Peroxisome

When encountering stresses, plants respond within several seconds to minutes -, and the genes involved in this rapid response are vital, as they initiate transcriptional and metabolic responses leading to improved survival. Until now, our knowledge of the nature and function of the rapid and systemic responses to abiotic stresses in plants has been limited and only a few key genes involved in this process were isolated and functionally characterized (Kollist et al., 2018). *SUPA* expression rapidly responds to abiotic stresses, within 15 min, which may lead to the broad transcriptomic changes. Whereas elevated *SUPA* expression, does not directly change the downstream gene expression of other master stress-responsive transcription factors such as DREBs or WRKYs as it is localized in peroxisomes. Peroxisomes are an important source of signaling molecules that function in plant growth and development under both normal and stress conditions (Hu et al., 2012; Sandalio and Romero-Puertas, 2015). It has been reported that peroxisomes could rapidly sense environmental changes and adjust the level of ROS accordingly, which then enables ROS-mediated nuclear gene expression, although the molecular mechanisms involved in these processes are still unknown (Queval et al., 2007; Mittler et al., 2011). *SUPA* maybe involve in this process, as it rapidly responds to abiotic stresses, induces the ROS accumulation and the subsequent gene expression. However, the question still remains of how SUPA protein affects ROS level at molecular level.

### ROS Is Accumulated by *SUPA* Overexpression, Which Confers Differing Plant Stress Tolerance Abilities in Different Species

ROS accumulation mediated by the overexpression of *SUPA* could be observed in both Arabidopsis and poplar, and they share some similar phenotypes such as curved leaves and smaller plant size when grown under normal conditions. However, their responses to stresses are opposite. Transgenic Arabidopsis had enhanced tolerance to abiotic stress whereas transgenic poplars had a decreased tolerance to salt, and increased tolerance to water deficit. Several possibilities may lead to these results. Firstly, ROS could act as signaling molecular only at low concentration, while causing oxidative stress at high concentration, and different plant species may respond to similar concentrations of ROS differently. In the future, more species overexpressing the *SUPA* gene will be used to investigate plant tolerance changes to abiotic stress to test this hypothesis. Secondly, ROS production and scavenging are both important in controlling ROS levels. In Arabidopsis, ROS could effectively be induced by SUPA accumulation, although there must be a regulatory mechanism that could trigger the cessation of *SUPA* expression when not required so that ROS levels can return to normal. In poplar, such anti-SUPA-function mechanisms may not work effectively, leading to the over-accumulation of ROS and reduced plant stress tolerance. Thirdly, maybe there were some unknown mechanism that contribute to the observed phonotype exist in poplar. Further detailed functional analysis of SUPA protein will be performed in the future.

### Important Factors That May Contribute the SUPA-OX Phenotypes

In our study, 18 transcription factors with at least twofold change in expression were identified (Supplementary Table 7). Remarkably, most of these transcription factors were reported to be involved in plant stress responses (Wang et al., 2016; Zhu, 2016). Overexpression of some transcription factors such as *WRKY30* (Scarpeci et al., 2013), *ZFP1* (Tang et al., 2013), *HSFB3* (Scharf et al., 2012) effectively enhanced plant tolerance to abiotic stresses, and therefore the enhanced expressions of these transcription factors in both *SUPA-OX* lines may also contribute to the plant stress resistance phenotypes. Moreover, 77 stress responsive genes were identified among the DEGs (Supplementary Table 8) and 24 of these genes were involved in plant defense response (Supplementary Table 9), indicating the potential role of *SUPA* in the plant defense response.

*SUPA*-overexpression lines display early flowering phenotypes, and corroborating these results, we identified the following flower development related transcription factors with alternations in gene expression. *MAF4 (AT5G65070)* which was previously identified as a MADS-domain containing FLC paralog represses flowering (Gu et al., 2009, 2013), and overexpression of *MAF4* leads to delayed flowering (Ratcliffe et al., 2003). *AHL22 (At2g45430)* which encodes a nuclear localized AT-hook domain containing protein binds to an AT-rich sequence stretches in the FT locus (Yun et al., 2012). Overexpression of *AHL22* leads to delayed flowering, while the alh22 mutant showed earlier flowering times compared with wild type (Xiao et al., 2009; Yun et al., 2012). In *SUPA* overexpression lines, both genes were significantly down-regulated (Supplementary Table 7) and those results correlated with the early flowering phenotype of the *SUPA-OX* lines.

*SUPA* overexpression leads to strong morphological alternations, and we identified 36 developmental related genes among the DEGs (Supplementary Table 10). Among them, 3 ethylene biosynthesis genes (*ACS1, ACS2, and ACS4*) and 6 auxin pathway genes (*LBD16, SAUR72, AT4G12980, WAG2, WAG1, SAUR72*) had significant changes in the overexpression lines, indicating both hormones may be involved in the *SUPA*-mediated phenotypes. 25 cell wall related genes were identified (Supplementary Table 11), indicating *SUPA* overexpression may change the cell wall properties and contribute to the observed phenotypes. The correlations between SUPA and these factors will be further investigated in the future.

**FIGURE 11:**
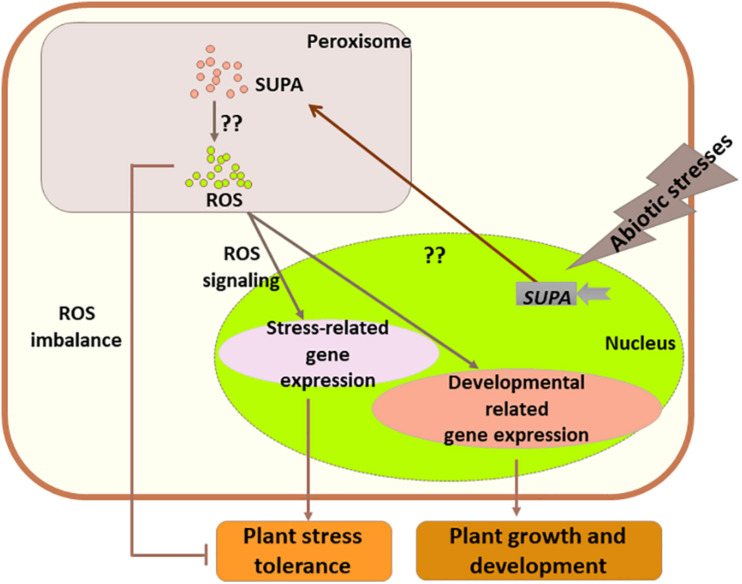
Working model for the role of *SUPA* in normal growth and stress conditions. Abiotic stresses rapidly activate the transcription of *SUPA*, and enhanced SUPA protein accumulation in peroxisomes, which leads to the increased level of ROS through unknown mechanisms. In some species such as Arabidopsis, the *SUPA*-mediated ROS acts as signaling component, and alters plant morphology and plant abiotic stress tolerance through the expression changes of the genes involved in plant stress response and development. In poplars, overexpression of *SUPA* results in higher ROS concentration than its tolerance threshold, which causes the imbalance of ROS level, and thereby reduced plant stress tolerance.

Based on our data, we hypothesize that in Arabidopsis the expression of *SUPA* could be rapidly activated by abiotic stress stimuli, and SUPA protein would accumulates in the peroxisome which would leads to the over-production of ROS through currently unknown mechanisms. The increased levels of ROS act as the signaling molecule to activate the stress responsive genes and thereby improve Arabidopsis tolerance to abiotic stresses. In transgenic poplar, a more complex mechanism is suggested, as SUPA-overexpression also leads to the enhanced ROS level, which, in addition to the ROS signaling effect leading to poplar tolerance to water deficit, also causes oxidative stresses and reduces tolerance to salt stress (Figure 11). These findings will largely improve our understanding of plant physiology, and the further study of regulatory mechanisms of *SUPA* gene expression and the mechanisms of action of SUPA protein will enable a greater and more complete understanding of its molecular functions.

## Data Availability Statement

The data were deposited in NCBI Sequence Read Archive (SRA, http://www.ncbi.nlm.nih.gov/sra) with accession number PRJNA634204.

## Author Contributions

QZ conceived this project, designed the experiments, and interpreted the results. CC and WW performed the experiments. SY, CW, and MX helped to collect and analyze the data. ZZ helped to analyzed the RNA-seq data. QZ wrote the manuscript. All authors contributed to the article and approved the submitted version.

## Conflict of Interest

The authors declare that the research was conducted in the absence of any commercial or financial relationships that could be construed as a potential conflict of interest.

## Footnotes

1 http://www.ncbi.nlm.nih.gov/sra

2 http://www.plantgdb.org/PtGDB/

## Supplementary Material

Supplementary Figure 1 Phylogenetic analysis of *SUPA* and its homologues. **(A)** Phylogenetic analysis of SUPA and proteins shared the highest similarities in Arabidopsis. The protein sequences of *SUPA* (*AT5G65300*) and proteins shared the highest similarities (*AT1G35210*, *AT1G72240*, and *AT1G22470*) were downloaded from TAIR, and the neighbor-joining method of the MEGA5 program was used to construct the phylogenetic tree. Bootstrap values from 1000 replicates are indicated at each branch. **(B)** Phylogenetic analysis of SUPA and its homologues in poplar. The protein sequences of *SUPA* (*AT5G65300*) and proteins shared the highest similarities (*POPTR_0007s05020.1*, *POPTR_0005s07300.1*, and *POPTR_0005s17370.1*) were downloaded from poplar genome database (http://www.plantgdb.org/PtGDB/), and the neighbor-joining method of the MEGA5 program was used to construct the phylogenetic tree. Bootstrap values from 1000 replicates are indicated at each branch. **(C)** Alignment of the amino acid sequence of SUPA and proteins shared the highest similarities. Alignment profiles of SUPA (AT5G65300) and proteins with the highest (AT1G35210, AT1G72240, AT1G22470) with Clustal-W program are shown. The shading indicates identical and conserved amino acid residues. The three conserved domains are underlined (Domain I-Domain III). **(D)** Alignment of the amino acid sequence of SUPA and proteins shared the highest similarities. Alignment profiles of SUPA (AT5G65300) and proteins with the highest (*POPTR_0007s05020.1*, *POPTR_0005s07300.1*, and *POPTR_0005s17370.1*) with Clustal-W program are shown. The shading indicates identical and conserved amino acid residues. The three conserved domains are underlined (Domain I-Domain III).

Supplementary Figure 2 The transcripts of *SUPA* homologues rapidly respond to ABA and abiotic stresses. The expression of *AT1G22470*, *AT1G72240*, and *AT1G35210* in response to ABA and various abiotic stresses were analyzed by qRT-PCR. 7-day-old seedlings grown on AM+ medium were treated with 150 mM NaCl, 1 μM, 4 °C, and 300 mM Mannitol. Samples were collected at the indicated time after the initiation of treatment. Total RNA was extracted from whole seedlings, and real-time PCR analyses were performed with the gene specific primers listed in Supplementary Table 8. The relative expression was calculated using Actin as an internal reference. The unstressed expression level was assigned a value of 1. Data represents the average of three independent experiments ± SE.

Supplementary Figure 3 Overexpression of *SUPA* leads to various phenotypic alterations in light but not in dark. **(A)** Phenotypes of seedlings grown in different types of light. Seeds were germinated and grown under the light indicated on the left, the results were recorded at day 7. Bar = 0.5 mm. **(B–G)** Statistical analysis of the hypocotyl length and petiole grown in various light conditions. Hypocotyl length under white light **(B)**; Petiole length under normal light **(C)**; Hypocotyl length in far red light **(D)**; Petiole length in far red light **(E)**; Hypocotyl length of blue light **(F)**; Petiole length under blue light **(G)**; Hypocotyl length under dark **(H)**.

Supplementary Figure 4 Effects of *SUPA* overexpression on Arabidopsis flower growth. **(A)** Sepal phenotypes of wild type and three *SUPA*-overexpression lines at stage 12. Images were taken of 5-week old plants. Flowers were selected before opening and images were taken. Bar = 25 μm. **(B)** Carpel phenotypes of wild type and three *SUPA*-overexpression lines at stage 12. The sepals and petals from were carefully removed with forceps, and pictures of carpels were taken. Bar = 25 μm. **(C)** SEM images of the upper part of sepals at stage 15. The sepals and petals are open with the development of carpels, while the sepals of the *SUPA* overexpression lines keep closed. Bar=150 μm. **(D)** SEM images of the basal part of sepals at the stage 16. The basal parts of WT remain together, while in *SUPA* overexpression lines, the sepals rupture as the carpel develops. Bar=150 μm. **(E)** SEM images of the ovary at the stage 15. Bar=150 μm. **(F,G)** *SUPA-OX* lines have normal siliques after self-pollination. Pictures of the siliques grown from *SUPA-OX* lines without self-pollination **(F)**, and after self-pollination **(G)**.

Supplementary Figure 5 The *supa* mutant does not cause any phenotypic alternation under both normal growth and stress conditions. **(A)** *SUPA* gene in Arabidopsis. *SUPA* has one exon with a nucleotide length of 453 bp, the T-DNA fragment of *supa* mutant (SALK_069313) was inserted 55 bp from start code, as shown in the schematic. **(B)** RT-PCR verification of *supa* mutant. 7-day old seedling was used for RT-PCR analysis with *SUPA* gene specific primers, with the expression level of *Actin* as the control. **(C)** *supa* mutant is comparable to wild type in both normal growth and stress conditions. Seeds of wild type (WT) and *supa* mutant were sown on AM+ medium **(A)**, or AM+ medium containing 1 μm ABA **(B)**, 150 mM NaCl **(C)**, 300 mM mannitol **(D)**, photos were taken 7 days after stratification, and the representative images from three independent experiments are shown here. Bar = 0.3 cm. **(E)** 1-month old wild type and *supa* mutants grown on the soil.

Supplementary Figure 6 The phenotypes of *SUPA*, *SUPA-HA*, and *SUPA-GFP* overexpression lines. Phenotypes of seedlings of WT, *SUPA-OX1* and *SUPA-HA*. Seeds were germinated and seedlings were grown in the white light, and 7-day old seedlings were imaged. **(A)** Cotyledon and hypocotyl; **(B)** Seedlings. Bar = 0.5 mm.

Supplementary Figure 7 SUPA protein is not co-localized with Lysosome and Mitochondria. *Arabidopsis* expressing the *SUPA-HA* construct was crossed with the Lysosome marker 35S::GFP-8a or Mitochondria marker Mt-RK expressing *Arabidopsis* line. The Lysosome marker (up panel) and the Mitochondria marker (low panel) of the 7-day old seedlings from the F1 progeny were used for immunostaining with anti-HA (green) and anti-RFP (red) antibody. Pictures were taken using a Zeiss LSM 880 confocal scanning microscopes with Airyscan. In the up panel, Bar = 5 μm, and in the low panel, Bar = 2 μm.

Supplementary Figure 8 The concentration of H_2_O_2_ and the level of T-AOC under different stress conditions in the Arabidopsis. Quantitative measurements of H_2_O_2_ content **(A)** and T-AOC level **(B)** in WT and *SUPA-OX* lines under different stress conditions in Arabidopsis. Data represent the mean ± SE of three independent measurements. ***p* < 0.01.

Supplementary Figure 9 Ectopic expression of *SUPA* in poplar alters the content of ROS. **(A)** NBT staining (upper panels) and DAB staining (lower panels) show the superoxide and H_2_O_2_ levels respectively. The 3^rd^ leaves from the top of the 1-month old poplar were used for staining. The experiments were repeated at least 3 times, and representative data is shown here. Bar = 1.5 cm. **(B,C)** Quantitative measurements of H_2_O_2_ content **(B)** and T-AOC level **(C)** in WT and *SUPA-OX* lines under normal conditions in poplar. Data represent the mean ± SE of three independent measurements. ***p* < 0.01.

Supplementary Figure 10 Overexpression of *SUPA* in poplar reduces plant salt tolerance. Wild type poplars and three *SUPA*-overexpression transgenic lines were grown on 1/2 MS medium with various concentration of NaCl (50 mM, 100 mM and 150 mM). The pictures before (Day 0) and after (Day 21) salt treatment were recorded, and representative data is presented. Three biological repeats were performed for all experiments.

Supplementary Table 1 List of all the DEGs.

Supplementary Table 2 GO annotations of the DEGs between wildtype and SUPA-overexpression lines.

Supplementary Table 3 GO annotations of the DEGs between wildtype and SUPA-overexpression lines especially in SUPA-OX1.

Supplementary Table 4 GO annotations of the DEGs between wildtype and SUPA-overexpression lines especially in SUPA-OX2.

Supplementary Table 5 List of the peroxisome localized genes among DEGs between wildtype and SUPA-overexpression lines.

Supplementary Table 6 GO annotations of the DEGs between wildtype and SUPA-overexpression lines that localized in peroxisomes.

Supplementary Table 7 List of the transcription factors among the DEGs between wildtype and the SUPA-overexpression lines.

Supplementary Table 8 List of the stress related genes among DEGs between wildtype and SUPA-overexpression lines.

Supplementary Table 9 List of the defense related genes among DEGs between wildtype and SUPA-overexpression lines.

Supplementary Table 10 List of the developmental related genes among DEGs between wildtype and SUPA-overexpression lines.

Supplementary Table 11 List of the cell wall related genes among DEGs between wildtype and SUPA-overexpression lines.

Supplementary Table 12 List of primers used for analysis.
