# Supplementary material for: Overexpression of a Novel Arabidopsis Gene SUPA Leads to Various Morphological and Abiotic Stress Tolerance Alternations in Arabidopsis and Poplar
Source: Front Plant Sci. 2020 Nov 12;11:560985. doi: 10.3389/fpls.2020.560985 (PMC7688997; doi:10.3389/fpls.2020.560985)

Supplemental Figure 1. Phylogeny analysis of *SUPA* and its homologues

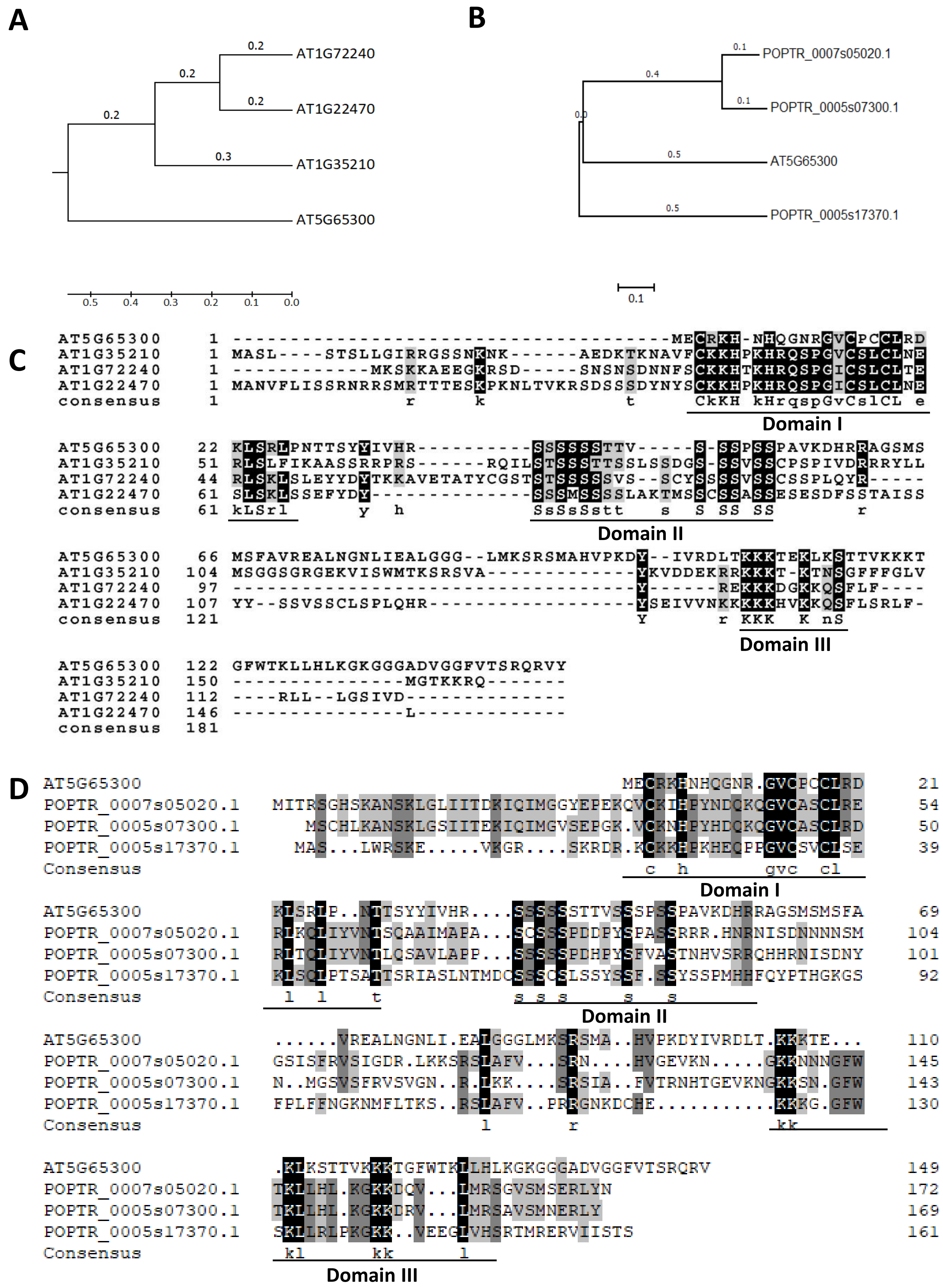

Supplemental Figure 2. The transcripts of *SUPA* homologues rapidly response to abiotic stresses

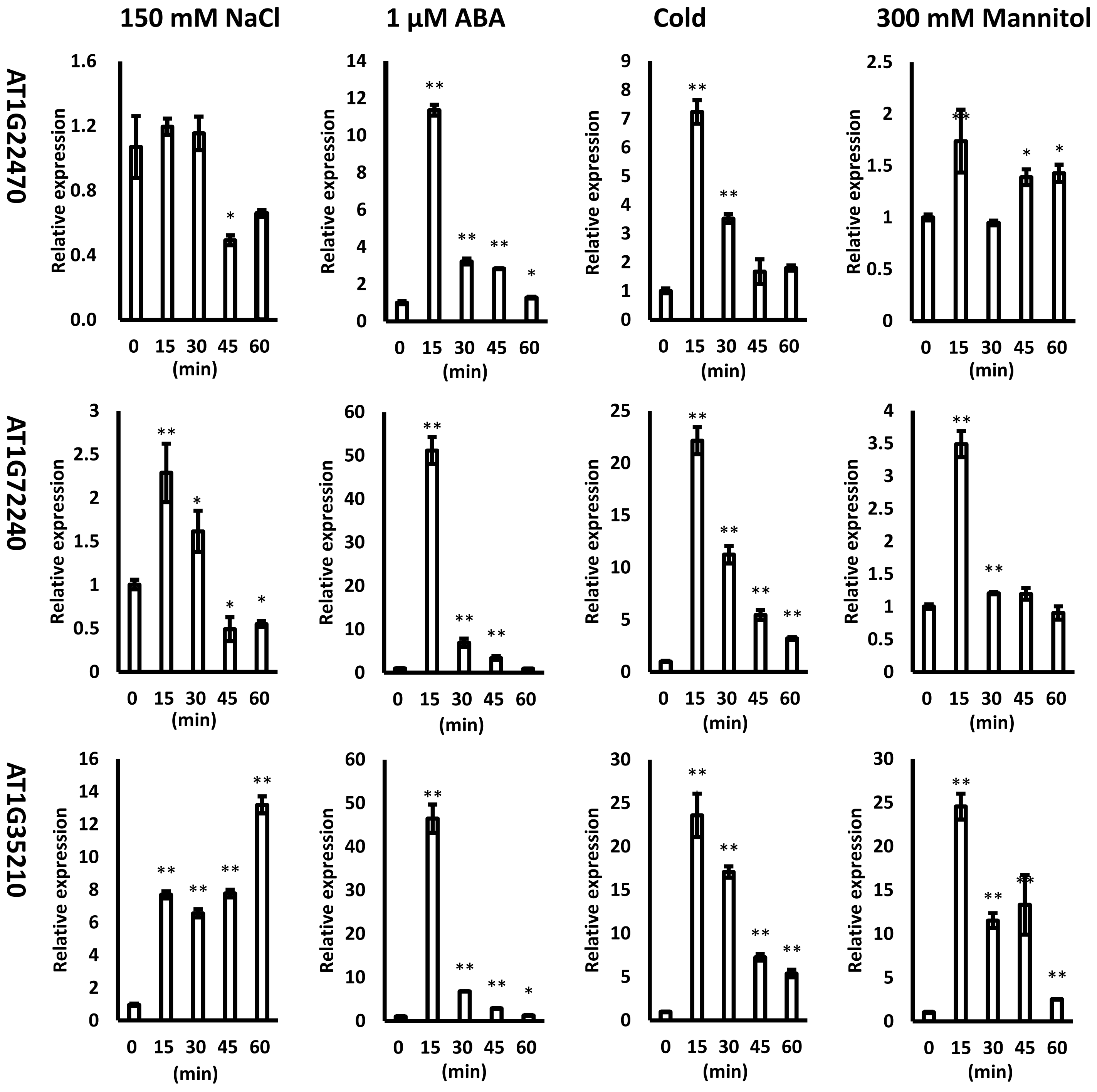

Supplemental Figure 3. Overexpression of *SUPA* leads to various phenotypic alternation in light but not in dark

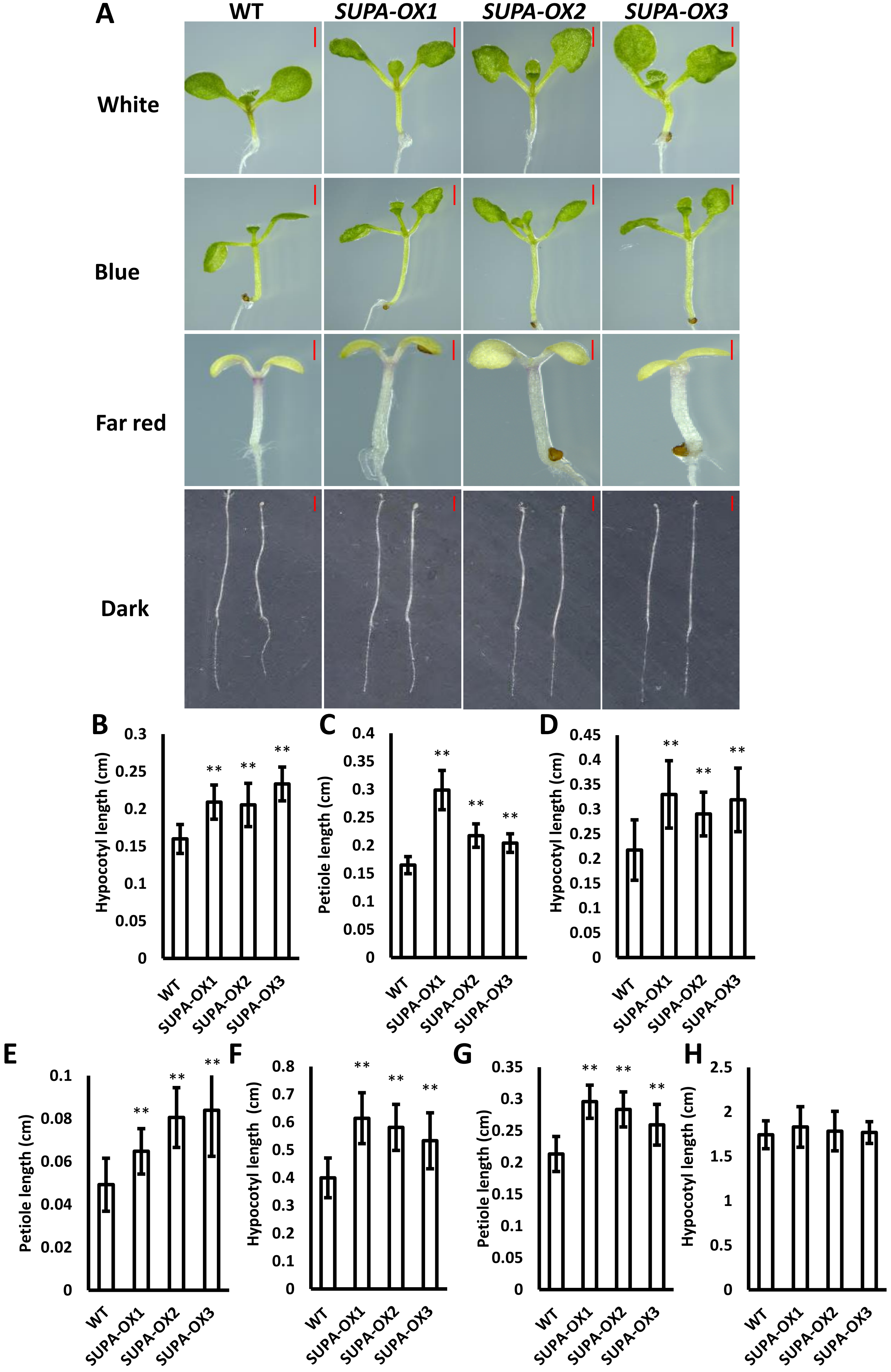

Supplemental Figure 4. Effects of *SUPA*-overexpression on Arabidopsis flower development

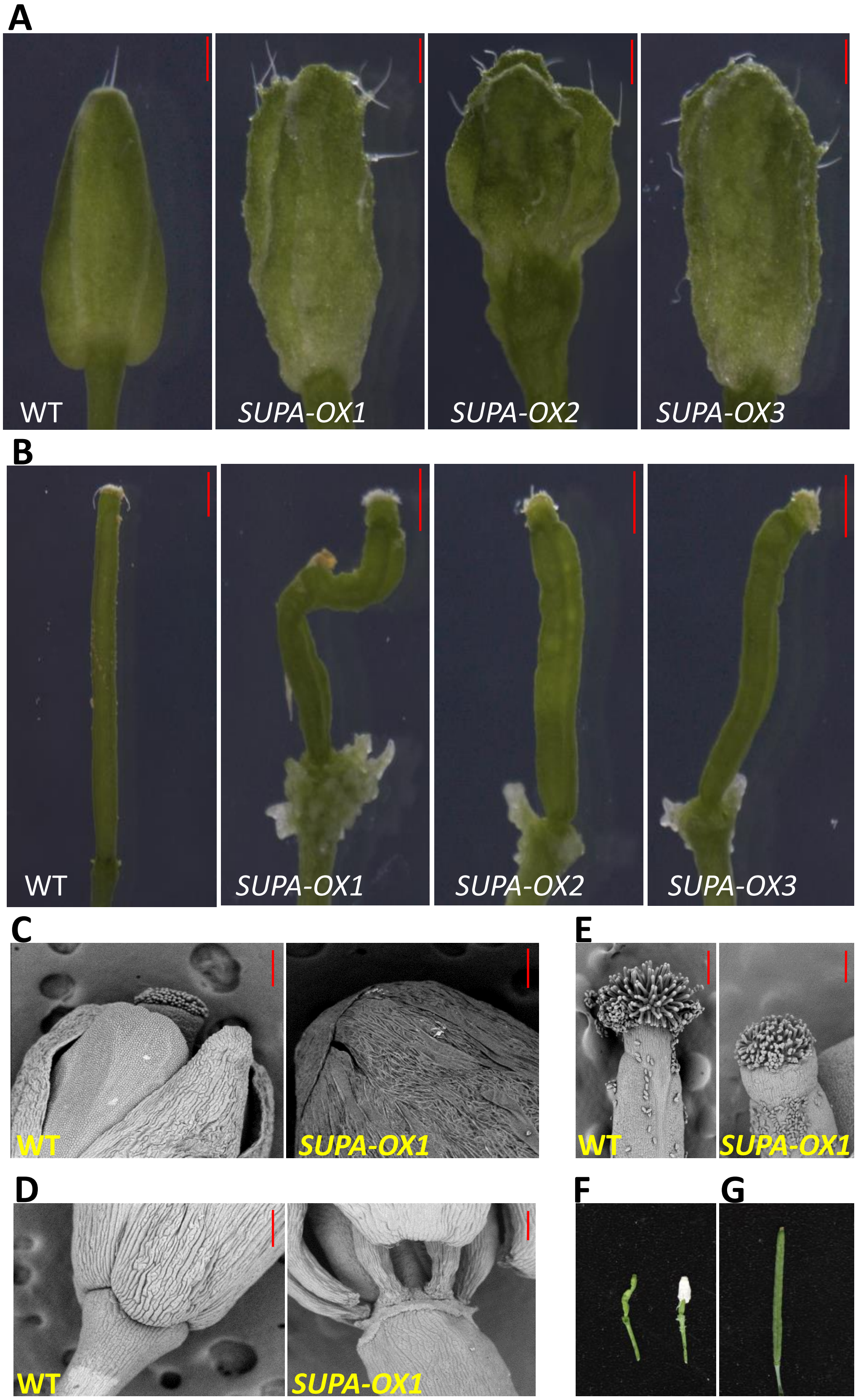

Supplemental Figure 5. ***supa*** **not causes any phenotypic alternation under both normal growth and stress conditions**

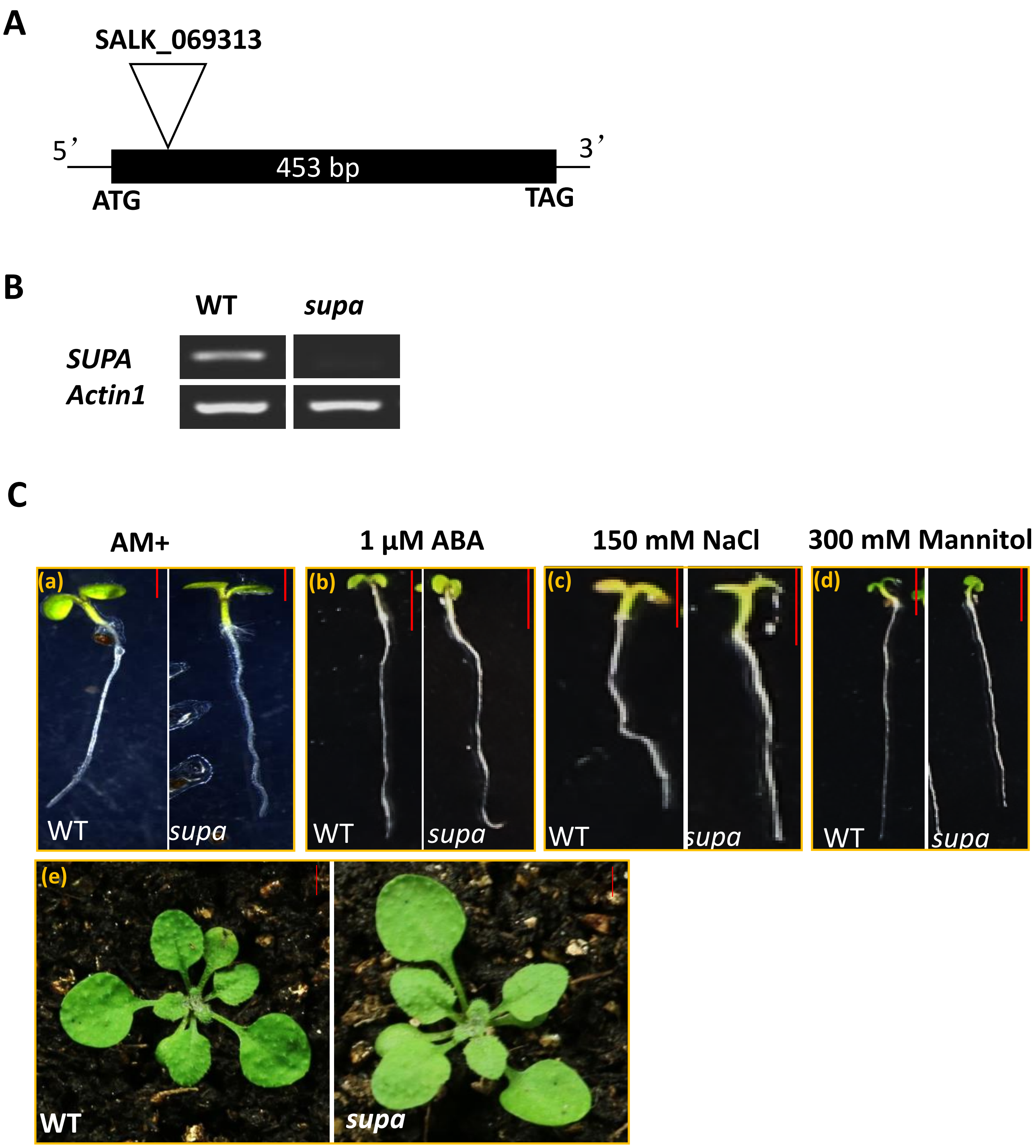

Supplemental Figure 6. The phenotypes of *SUPA*, *SUPA-HA*, and *SUPA-GFP* overexpression lines

A

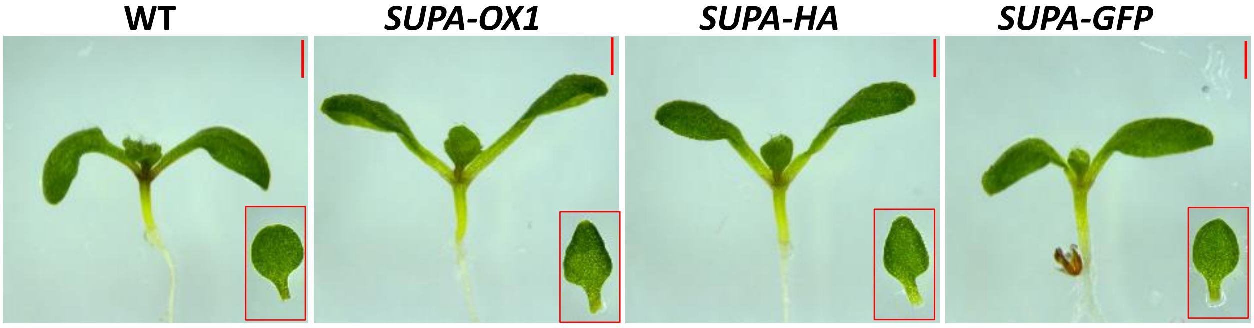

B

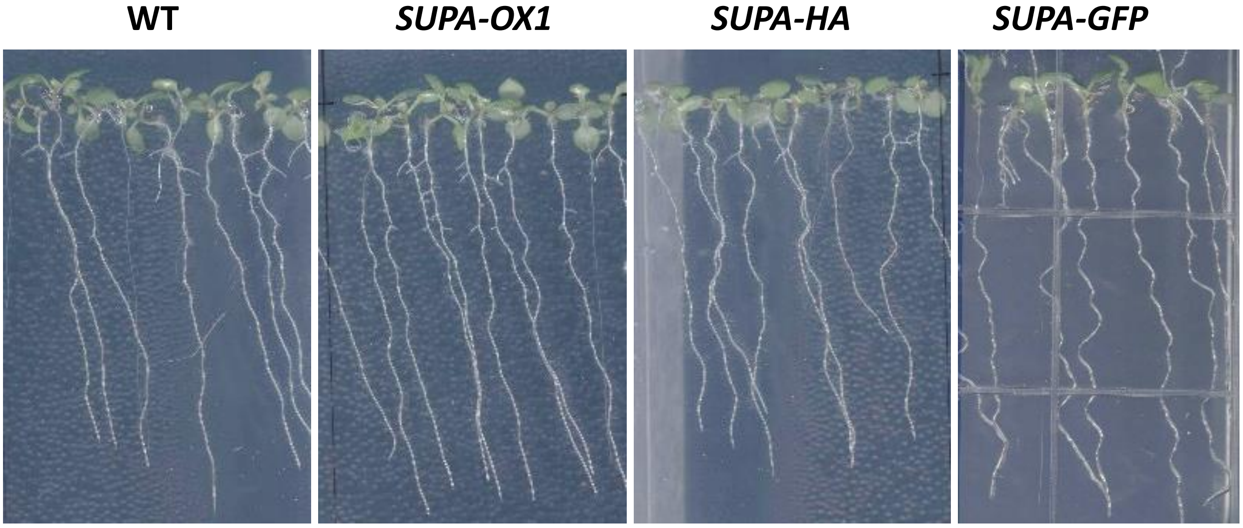

Supplemental Figure 7. SUPA protein is not co-localized with Lysozyme and Mitochondria.

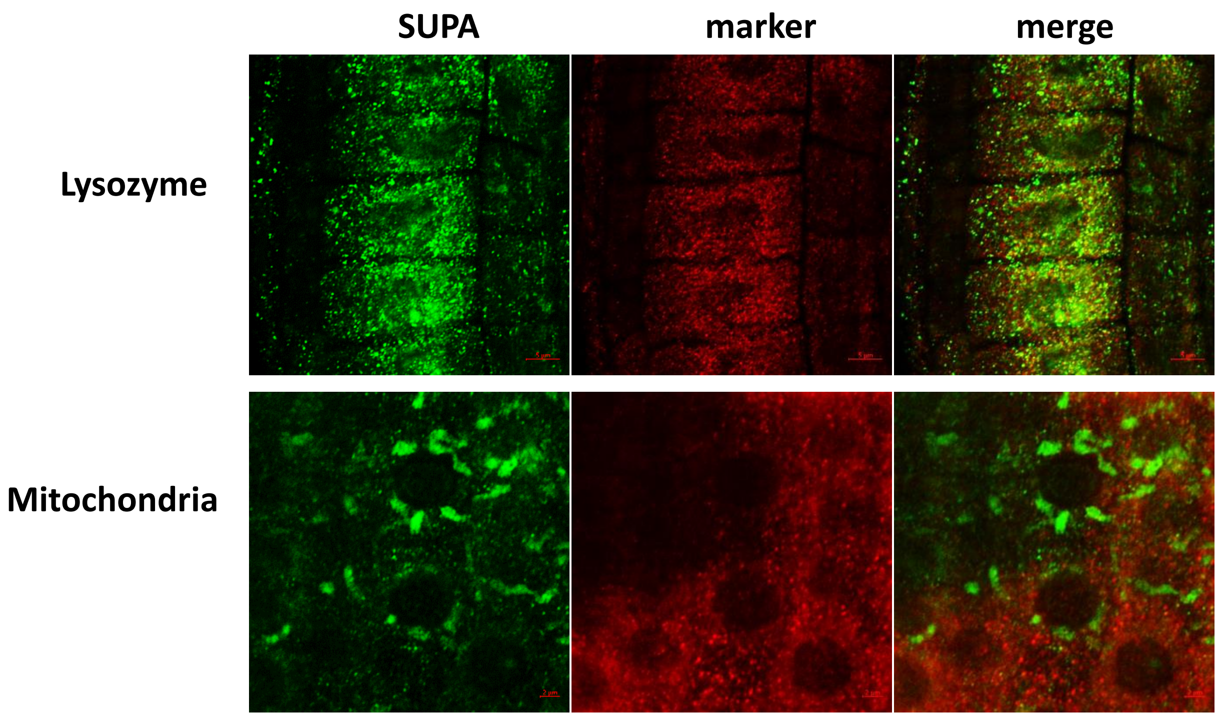

Supplemental Figure 8. The concentration of H_2O_2 and the level of T-AOC under different stress conditions in the Arabidopsis.

A

B

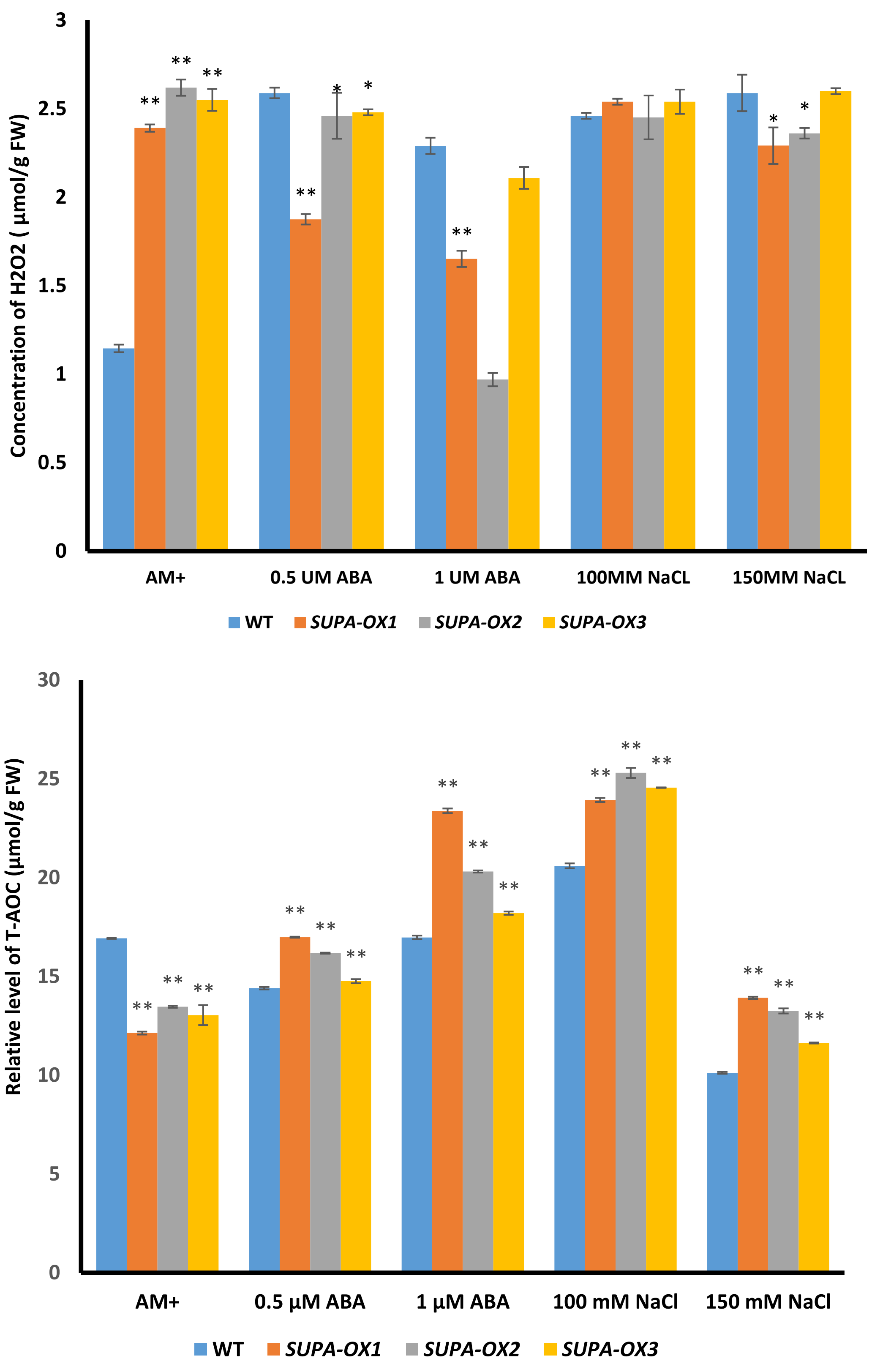

Supplemental Figure 9. Overexpression of *SUPA* alters the content of ROS in the leaves of transgenic poplar

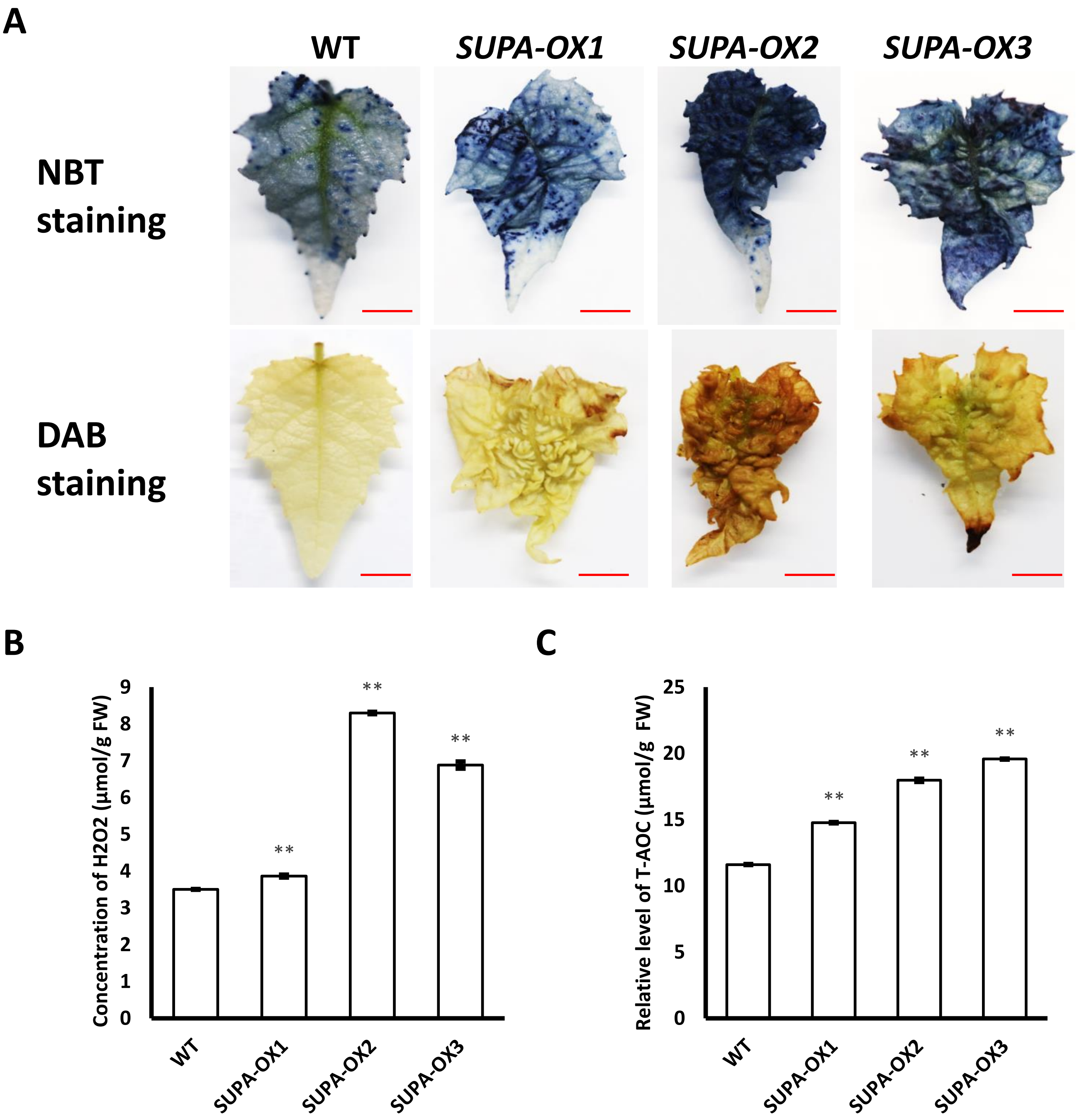

Supplemental Figure 10. Overexpression of *SUPA* in poplar reduces plant salt tolerance

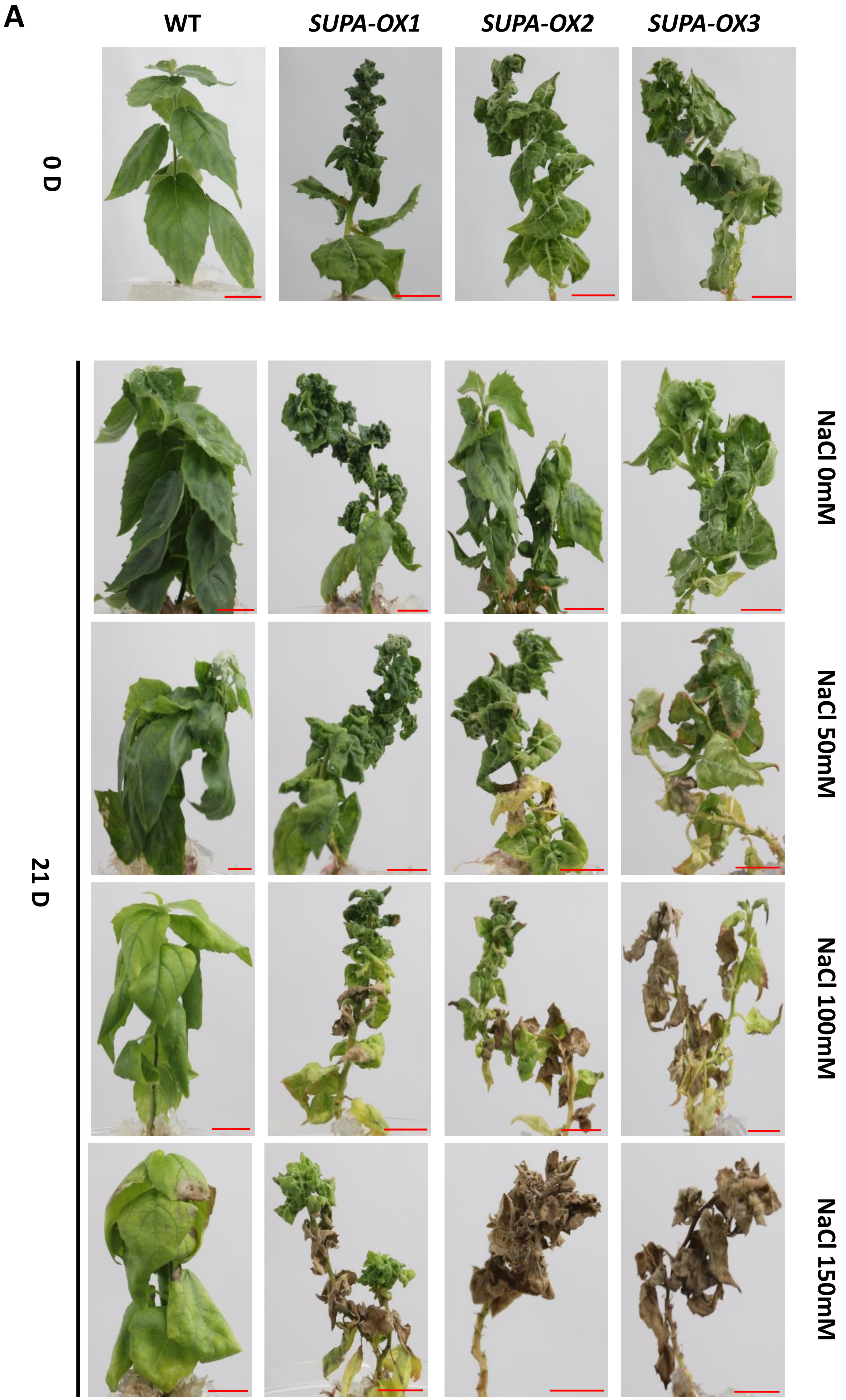

Supplement: Supplementary Figure 1 — Phylogenetic analysis of SUPA and its homologues. (A) Phylogenetic analysis of SUPA and proteins shared the highest similarities in Arabidopsis. The protein sequences of SUPA (AT5G65300) and proteins shared the highest similarities (AT1G35210, AT1G72240, and AT1G22470) were downloaded from TAIR, and the neighbor-joining method of the MEGA5 program was used to construct the phylogenetic tree. Bootstrap values from 1000 replicates are indicated at each branch. (B) Phylogenetic analysis of SUPA and its homologues in poplar. The protein sequences of SUPA (AT5G65300) and proteins shared the highest similarities (POPTR_0007s05020.1, POPTR_0005s07300.1, and POPTR_0005s17370.1) were downloaded from poplar genome database (http://www.plantgdb.org/PtGDB/), and the neighbor-joining method of the MEGA5 program was used to construct the phylogenetic tree. Bootstrap values from 1000 replicates are indicated at each branch. (C) Alignment of the amino acid sequence of SUPA and proteins shared the highest similarities. Alignment profiles of SUPA (AT5G65300) and proteins with the highest (AT1G35210, AT1G72240, AT1G22470) with Clustal-W program are shown. The shading indicates identical and conserved amino acid residues. The three conserved domains are underlined (Domain I-Domain III). (D) Alignment of the amino acid sequence of SUPA and proteins shared the highest similarities. Alignment profiles of SUPA (AT5G65300) and proteins with the highest (POPTR_0007s05020.1, POPTR_0005s07300.1, and POPTR_0005s17370.1) with Clustal-W program are shown. The shading indicates identical and conserved amino acid residues. The three conserved domains are underlined (Domain I-Domain III). [file Data_Sheet_1.PDF]
