# Supplementary material for: Overexpression of a Novel Arabidopsis Gene SUPA Leads to Various Morphological and Abiotic Stress Tolerance Alternations in Arabidopsis and Poplar
Source: Front Plant Sci. 2020 Nov 12;11:560985. doi: 10.3389/fpls.2020.560985 (PMC7688997; doi:10.3389/fpls.2020.560985)
Supplement: Supplementary Table 12 — List of primers used for analysis. [file Table_12.DOCX]

**Table S12. List of primers used for analysis**

| **Primer** | **Sequence** | | **Purpose** | |
| --- | --- | --- | --- | --- |
| Salk_069313_LP | | TGGATCTTCCGGTTAGATTTG | | Mutant identification |
| Sslk_069313_RP | | GTTGCCGTGAAGTAACAAACC | | Mutant identification |
| attb1_*SUPA* | | GGGGACAAGTTTGTACAAAAAAGCAGGCTTAATGGAATGCAGAAAACAC | | Overexpression of *SUPA* |
| attb2_*SUPA*_noSTOP | | GGGGACCACTTTGTACAAGAAAGCTGGGTAATAAACTCGTTGCCGTGA | | Overexpression of *SUPA* |
| attb2_*SUPA*_STOP | | GGGGACCACTTTGTACAAGAAAGCTGGGTATTAATAAACTCGTTGCCG | | Overexpression of *SUPA* |
| attb1_*SUPA*pro | | GGGGACAAGTTTGTACAAAAAAGCAGGCTTACATCTTTTATCAACACAATCACTTT | | GUS |
| attb2_*SUPA*pro | | GGGGACCACTTTGTACAAGAAAGCTGGGTATTTTCTATCTTCTTCGTTCTCTTTT | | GUS |
| *SUPA*_qPCR_F | | ACAACCACCAAGGCAACAGAGGAG | | *SUPA* qPCR |
| *SUPA*_qPCR_R | | AGACGAAACGGTGGTGGATGAAGA | | *SUPA* qPCR |
| EF1α_qPCR_F | | ATTGACAGGCGGTCTGGTAAGGAA | | *Poplar* actin |
| EF1α_ qPCR_R | | AAACGACCAAGTGGAGGATACGCT | | *Poplar* actin |
| Actin_qPCR _F | | TGTATGCCAGTGGTCGTACCA | | *Arabidopsis* actin |
| Actin_qPCR _R | | CCAGCAAGGTCGAGACGAA | | *Arabidopsis* actin |
| AT1G22470_qPCR _F | | TGCGGACAACAACAGAATCA | | AT1G22470 qPCR |
| AT1G22470_qPCR _R | | GGAGATTGTCTGTGCTTCGGA | | AT1G22470 qPCR |
| AT1G72240_qPCR _F | | TGCAAGAAGCACACGAAACA | | AT1G72240 qPCR |
| AT1G72240_qPCR _R | | GCAATACGTCGCCGTTTCAA | | AT1G72240 qPCR |
| AT1G35210_qPCR _F | | AGCAGCTTCTTCACGTAGAC | | AT1G35210 qPCR |
| AT1G35210_qPCR _R | | AGTATCGTCGGCGATCAACA | | AT1G35210 qPCR |
| AT3G07040_qPCR _F | | TCCGGCAAGTCTTGAGCAAT | | Heat shock proteins QPCR |
| AT3G07040_qPCR _R | | ACGTTTAAGCGGGTATGGCA | | Heat shock proteins QPCR |
| AT5G63020_qPCR _F | | GGGCGGATGGGAGTTGATAG | | Heat shock proteins QPCR |
| AT5G63020_qPCR _R | | TTGCTGAACGCCATTCTTGC | | Heat shock proteins QPCR |
| AT4G19510_qPCR _F | | GTTCAGCGCTGGAGAGTCTT | | Heat shock proteins QPCR |
| AT4G19510_qPCR _R | | ACCAACCCTGTGGAATCGTC | | Heat shock proteins QPCR |
| AT2G14610_qPCR _F | | CTCGGAGCTACGCAGAACAA | | Heat shock proteins QPCR |
| AT2G14610_qPCR _R | | CCTCACTTTGGCACATCCGA | | Heat shock proteins QPCR |
| AT5G02490_qPCR _F | | TACTCTTGCGTCGGAGTGTG | | Heat shock proteins QPCR |
| AT5G02490_qPCR _R | | AACAGGGTTCATGGCGACTT | | Heat shock proteins QPCR |
| SGS3_qPCR _F | | GGAGGAGAGTGGGAGGTCAT | | Heat shock proteins QPCR |
| SGS3_qPCR _R | | AAGGAGGGCGAGATACAGGT | | Heat shock proteins QPCR |
| HSP70_qPCR _F | | CCTACTCTTGCGTCGGTGTT | | Heat shock proteins QPCR |
| HSP70_qPCR _R | | GTTGGTAGGGTTCATGGCGA | | Heat shock proteins QPCR |
| HSP90.1_qPCR _F | | TGGTGGATGCGATTGACGAA | | Heat shock proteins QPCR |
| HSP90.1_qPCR _R | | TCTCAACCTTGTCCCCGAGA | | Heat shock proteins QPCR |
| HSP23.6-MITO_qPCR _F | | CGACGAGGAGCGTTAGTCAA | | Heat shock proteins QPCR |
| HSP23.6-MITO_qPCR _R | | AGAGTGTCCTGCTCCAAAGC | | Heat shock proteins QPCR |
| AT1G53540_qPCR _F | | ATGTGGCAGCGTTCACAAAC | | Heat shock proteins QPCR |
| AT1G53540_qPCR _R | | ATTCTCCGGCAGCCTAAACC | | Heat shock proteins QPCR |
| HSP17.4_qPCR _F | | GACGTGCCTGGGCTTAAGAA | | Heat shock proteins QPCR |
| HSP17.4_qPCR _R | | ACCGACAACACCCCATTCTC | | Heat shock proteins QPCR |
| HSP17.6A_qPCR _F | | ACCAGCTGACGTTATCGAGC | | Heat shock proteins QPCR |
| HSP17.6A_qPCR _R | | GCCGCAGAGATCTTCTCCAA | | Heat shock proteins QPCR |
| AT1G07400_qPCR _F | | AACAGCATCTTCGACCCGTT | | Heat shock proteins QPCR |
| AT1G07400_qPCR _R | | TCTTGAGCACACTGTCGTCC | | Heat shock proteins QPCR |
| AT3G03820_qPCR _F | | GCTCAGTAGCAGGAACGAGG | | Heat shock proteins QPCR |
| AT3G03820_qPCR _R | | AGGACAAGGGATCGTCAAGC | | Heat shock proteins QPCR |
